# Biomimetic Gradient Porous Core–Shell Fibers with Enhanced Gas Sensing for CO-Temperature Dual-Mode Early Fire Warning

**DOI:** 10.1007/s40820-026-02292-8

**Published:** 2026-08-03

**Authors:** Lele Huang, Xingyu He, Jianan Jiang, Xiaoqian Li, Mi Zhou, Md Hasib Mia, Min Hong, Hualing He, Siqi Huo, Zhicai Yu

**Affiliations:** 1 State Key Laboratory of New Textile Materials and Advanced Processing, Hubei Key Laboratory of Biomass Fibers and Eco-Dyeing & Finishing, School of Textile Science and Engineering, Wuhan Textile University, Wuhan, 430200 People’s Republic of China; 2 Key Laboratory of High Performance Fibers & Products, Ministry of Education, College of Materials Science and Engineering, Donghua University, Shanghai, 201620 People’s Republic of China; 3 Centre for Future Materials, School of Science, Engineering and Digital Technologies, University of Southern Queensland, Springfield Central, 4300 Australia

**Keywords:** Early fire warning, Bimodal sensing fiber, Gradient porous, Coaxial wet spinning, CO-temperature detection

## Abstract

Early fire detection systems that are highly sensitive are essential for reducing the impact of fire disasters. However, their development still faces significant challenges due to the lack of capability for simultaneous monitoring of both temperature and gas. Herein, we propose a facile coaxial wet-spinning strategy to fabricate a dual-parameter fiber sensor capable of simultaneously detecting carbon monoxide (CO) and temperature for early combustion warning. The resulting core–sheath structured fiber consists of a CO sensing sheath made of SnO_2_/In_2_O_3_ heterojunction/aramid nanofiber (ANF)/silver nanowire composite with biomimetic gradient pores, an ANF isolation layer, and a temperature sensing core composed of MXene. The gradient porous sheath constructed by gradient-induced phase separation technology exhibits gradually decreasing pore sizes from outer (> 10 *μ*m) to inner (< 3 *μ*m) regions. This structure demonstrates a significant enhancement in the fiber sensor’s sensitivity to CO, achieving a 15% higher response compared to non-gradient porous structures (*ΔR/R*_*0*_ = *0.95%/ppm*; detection limit of 10 ppm), with the response time reduced to 19.28 s, surpassing the response speed of most fire-warning fibers. Additionally, this fiber sensor can rapidly monitor abnormal temperature increases, enabling flame alarm functionality within 3 s. It also achieves precise real-time temperature detection within the range of 50–300 °C, exhibiting high sensitivity (20.6 *μ*V K^−1^) and a strong linear correlation (*R*^*2*^ = 0.99). This work highlights the significant potential of gradient pore in enhancing CO sensing and offers a novel perspective for the design of ultrafast early fire-warning fiber sensors.

**Supplementary Information:**

The online version contains supplementary material available at 10.1007/s40820-026-02292-8.

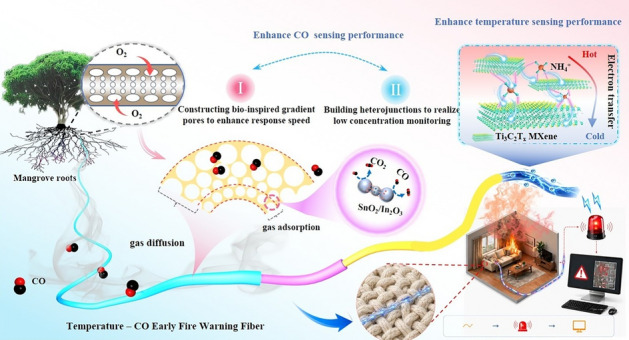

## Highlights

- A biomimetic gradient porous core–shell fiber with enhanced gas-sensing capabilities for CO-temperature early fire warning is fabricated via a coaxial wet-spinning technology.
- The gradient porous sheath with SnO_2_/In_2_O_3_ heterojunction endows enhanced CO gas-sensing performance with high sensitivity, low detection limit and improves the CO respond of by 15%.
- The CO-temperature dual-mode sensing fiber integrated with a wireless early fire-warning system achieves a rapid respond to fire in ~3 s and detects 10 ppm CO gas within 19 s.

## Introduction

Fire plays a dual role in the advancement of human civilization, acting as both a crucial driver of development and a potential source of devastating disasters. The construction site fire in Istanbul, Turkey, in 2025, and the late-year commercial building fire in Dhaka, Bangladesh, in 2024, both resulted in significant tragedies, claiming dozens of lives due to ineffective control of initial ignition sources, compounded by issues such as obstructed evacuation routes. These disasters not only resulted in immediate loss of life and property but also caused long-term and profound environmental damage [1–3]. Such incidents often originate from inconspicuous ignition sources that, if not promptly detected, can rapidly escalate into uncontrollable blazes. Therefore, the development of efficient and reliable early fire-warning technologies has become an urgent global priority for ensuring public safety and mitigating disaster losses [4–6]. Currently, widely used early fire-warning devices, including traditional smoke detectors and infrared thermal sensors, primarily detect fires by monitoring signals such as smoke particles or changes in environmental temperature [7, 8]. However, these technologies have inherent limitations: their physical structures are typically fixed, lacking flexibility in installation and layout. Furthermore, they heavily depend on a continuous and stable power supply, as any power outage or instability can disrupt warning functions, thereby undermining system reliability and practical deployability. To address these challenges, the scientific community has focused on emerging nanomaterials and flexible sensing technologies [9]. High-sensitivity fire-warning sensors based on graphene oxide (GO) or metal oxide semiconductors (MOS) have been developed to detect environmental temperature changes during the early stages of a fire through microscopic structural engineering [10–12]. For instance, some studies have functionalized graphene oxide to enhance its sensitivity to minute temperature fluctuations, while other teams have employed two-dimensional material composites such as Ti_3_C_2_T_X_ MXene to specifically detect hallmark gases released during the initial stages of a fire [13]. These intelligent fire-warning sensors exhibit response speeds that significantly surpass those of traditional technologies [14, 15]. However, the reliance on a single-signal detection model still poses significant challenges for accurate early fire warning in practical applications. It is well known that fire is a complex process involving heat release, particulate generation, and the emission of various gases [16]. A single temperature sensor is insufficient for effectively detecting smoke released during combustion and the characteristic gases produced by smoldering fires. Similarly, a single smoke or gas sensor may not adequately detect rapidly heating flames that produce low smoke. Therefore, dual-mode sensing technologies capable of simultaneously monitoring multiple critical parameters are considered a more promising solution.

A dual-parameter early fire-warning sensor is designed to simultaneously capture two core characteristic signals of early fires: the abnormal increase in ambient temperature and the sharp rise in the concentration of early fire marker gases, such as carbon monoxide (CO) [17, 18]. CO is a typical product of the incomplete combustion of carbonaceous materials, particularly during the smoldering phase, and its concentration in the air significantly increases in the early stages of a fire, making it a critical early fire-warning indicator. Currently, flexible fiber-based fire-warning sensors have garnered significant attention due to their conformability, ease of integration, and ability to adhere to complex surfaces [19–21]. This characteristic allows fiber-based fire-warning sensors to be positioned closer to potential ignition points, enabling faster and more sensitive monitoring of early stage fires. However, integrating temperature and gas-sensing functions into a single sensing fiber and achieving precise signal differentiation and decoupling of signals remains a challenge. The primary difficulty lies in the issue of cross-sensitivity: when the sensor is subjected to composite stimuli, such as simultaneous temperature increases and CO gas exposure, the resulting electrical signal patterns may be similar or overlapping [22, 23]. Among various temperature and gas-sensing materials, thermoelectric materials based on the Seebeck effect and metal oxide semiconductor materials with gas-sensing capabilities stand out for their ability to provide crosstalk-free signals during both temperature sensing and gas-sensing processes [24–26]. This distinction arises because thermoelectric materials based on the Seebeck effect can spontaneously and continuously convert temperature differential signals into voltage signals, thereby distinguishing themselves from the resistance signals generated by semiconductors when stimulated by gases [27]. In addition, reasonable structural design is also crucial for achieving effective separation of multi parameter signals. Among them, the coaxial structure can build independent sensing channels inside the fiber, which can, respectively, carry different functional materials and their corresponding sensing signals, so as to effectively distinguish the response behavior of different sensing units, reduce the possibility of mutual interference and coupling between signals, and provide a structural basis for achieving stable and accurate dual-parameter collaborative monitoring [28–30]. Among all thermoelectric materials, the two-dimensional transition metal carbon/nitride Ti_3_C_2_T_X_ MXene has emerged as one of the ideal choices for temperature monitoring due to its exceptional thermoelectric performance [31–35]. However, MXene’s inherent tendency to self-stack reduces the number of exposed active sites and hinders the formation of a stable conductive network in fiber substrate [36]. However, through ionic crosslinking, the intrinsic self-stacking tendency of MXene can be effectively mitigated, allowing MXene nanosheets to establish a stable spatial network structure in fiber [37, 38]. This method enables MXene to maintain a robust conductive network within fiber substrate, thereby optimizing the utilization of their thermoelectric effects in temperature sensing applications. SnO_2_ is a cost-effective, stable, and tunable n-type semiconductor that is suitable for CO sensing [39]. However, it suffers from poor selectivity due to cross-sensitivity to H_2_, CH_4_, ethanol, and NO_2_. The combination of SnO_2_ with In_2_O_3_ results in an *n*–*n* heterojunction that enhances the sensitivity to CO through the formation of a thicker electron depletion layer and improved resistance change in a CO atmosphere [40, 41]. Nevertheless, the dense fiber structure restricts gas diffusion, thereby reducing the effective interaction between CO and the SnO_2_/In_2_O_3_ composite, which in turn diminishes its intrinsic sensing performance. At present, achieving high-sensitivity CO detection within fiber while maintaining the material’s inherent sensing capabilities remains a significant challenge.

To achieve precise monitoring of CO gas concentration signals in fiber systems, meticulous design of fiber microstructures is essential. Biological hierarchical porous structures provide multiscale-ordered pores that offer a significant specific surface area, optimizing gas diffusion and recognition through the presence of macropores, mesopores, and micropores. Macropores facilitate rapid gas transport, mesopores enhance adsorption capacity and increase the density of reaction sites, while micropores improve gas selectivity through confinement effects. Similarly, natural mangroves thrive in marsh environments, where the presence of marshes limits the oxygen available to their roots. Consequently, mangrove roots have evolved an ingenious gradient porous structure that transitions from large pores on the epidermal layer to micropores internally. The large pores in the epidermal layer increase its porosity, thereby enhancing the diffusion efficiency of oxygen into the root interior. Concurrently, the internal micropores create a substantial specific surface area, providing additional sites for oxygen adsorption. This ingenious structural design significantly improves oxygen utilization efficiency. Constructing a gradient porous structure in fibers will enhance the diffusion and adsorption of gases from the environment into the interior of the fibers, allowing the gas-sensing material to fully contact the target gas and significantly improving the gas-sensing performance in sensing fibers. However, the construction of a gradient porous structure in fibers that transitions from external large pores to internal small pores remains a pressing challenge that must be addressed.

In this study, we present a facile coaxial wet-spinning strategy to fabricate a dual-parameter fiber sensor capable of simultaneously detecting CO and temperature for early fire warning. The resulting core–sheath fiber consists of a CO sensing sheath made of SnO_2_/In_2_O_3_ heterojunction/aramid nanofiber (ANF)/silver nanowire composite with biomimetic gradient pores, an ANF isolation layer, and a temperature sensing core composed of NH_4_^+^ crosslinked MXene, collectively referred to as the self-interference all-mode (SIAM) sensing fiber. Through the cross-linking effect of NH_4_^+^, the self-stacking tendency of MXene was overcome, thereby forming a continuous and stable three-dimensional conductive network structure with a Seebeck coefficient of 20.6 *μ*V K^−1^ in the core of SIAM sensing fiber. The ANF decoupling isolation layer establishes a crucial electrical insulation barrier that prevents electrical signal crosstalk between temperature and CO sensing, while also providing exceptional mechanical stability and thermal protection. The sheath layer comprises a SnO_2_/In_2_O_3_ composite embedded in an aramid fiber matrix with silver nanowires (Ag NWs), which bridge dispersed heterojunctions to form effective conductive pathways. The gradient porous sheath, fabricated via gradient-induced phase separation, features pore sizes decreasing from > 10 *μ*m (outer) to < 3 *μ*m (inner), enhancing CO gas diffusion into the fiber. This design enables high CO sensitivity with a response time of 19.28 s, a sensitivity 0.95%/ppm, and a detection limit 10 ppm, which improves by ~ 15% compared to non-gradient porous sheaths. Finally, the dual-mode sensing fiber integrated with a wireless early fire-warning system achieves a rapid respond to fire in ~ 3 s. The overall capabilities of resulting core–shell sensing fiber enhance its potential for use in early fire detection systems, paving the way for the development of reliable and sensitive fiber sensors that can monitor both temperature and specific gases simultaneously.

## Experimental Section

### Materials

Poly-p-phenylene terephthalamide (PPTA) fibers were supplied by Shanghai Mingxi Industrial Co., Ltd. Ti_3_AlC_2_ powder (200 mesh, ≥ 98.0%), indium oxide (In_2_O_3_, ≥ 99.9%), and lithium fluoride (LiF, purity ≥ 99%) were procured from Shanghai McLean Biochemical Co., Ltd. Silver nitrate (AgNO_3_), ethylene glycol, ethanol (≥ 95%), tert-butanol, ammonium chloride (NH_4_Cl), potassium hydroxide (KOH), hydrochloric acid (HCl), and dimethyl sulfoxide (DMSO) were provided by China Pharmaceutical Chemical Reagent Co., Ltd. Tin dioxide (SnO_2_, ≥ 99.95%) and poly(vinylpyrrolidone) (Mw = 1,300,000) were obtained from Aladdin Co., Ltd. Isopropanol was purchased from Shanghai Hushi Laboratory Equipment Co., Ltd.

### Preparation of ANF Spinning Dope

ANFs were prepared from PPTA fibers via a non-solvent-induced phase separation method [42]. Briefly, PPTA (1.8 g) and KOH (1.5 g) were added to DMSO (80 mL) in a round-bottom flask and magnetically stirred at 1000 rpm at room temperature. Subsequently, isopropanol (20 mL) was added to the mixture, facilitating isopropanol-assisted intercalation and exfoliation of the aramid fibers. This process resulted in a viscous, reddish-brown ANF spinning dope with a final concentration of 18 mg mL^−1^.

### Fabrication of SIAM Sensing Fiber

The shell-layer spinning dope for the SIAM sensing fibers was prepared by uniformly dispersing a SnO_2_/In_2_O_3_ composite in DMSO, followed by the addition of this mixture to the previously mentioned ANF spinning dope. Subsequently, a specified amount of Ag NWs was incorporated, and the mixture was thoroughly stirred. The SIAM sensing fibers were fabricated using a triaxial wet-spinning technique, employing a coaxial spinneret composed of three concentric needles: an 18 *G* needle (inner diameter: 0.84 mm) for the core, a 13 *G* needle (inner diameter: 1.8 mm) for the intermediate layer, and a 10 *G* needle (inner diameter: 2.70 mm) for the shell. The MXene dispersion, ANF dope, and the prepared shell-layer dope were injected into the core, intermediate, and shell channels of the spinneret, respectively. A graded phase separation strategy was adopted during the wet-spinning process to induce a gradient porous structure within the fibers. Specifically, a 50% (v/v) aqueous DMSO solution containing 5 wt% NH_4_Cl for crosslinking the core layer MXene was used as the first coagulation bath, while a 10% (v/v) aqueous acetic acid solution served as the second coagulation bath. The spinning dopes were extruded through the spinneret at flow rates of 25 *μ*L s^−1^ (shell), 20 *μ*L s^−1^ (intermediate), and 10 *μ*L s^−1^ (core), sequentially passing through the two coagulation baths. A constant draw ratio of approximately 1.1 was maintained during the spinning process. The as-spun fibers were immersed in a 20% (v/v) aqueous tert-butanol solution to remove residual water, collected, and subsequently freeze-dried to obtain the SIAM sensing fibers. For comparative analysis, a control fiber, designated as AM fiber, was prepared similarly using only MXene dispersion and ANF dope as the core and shell layers, respectively.

### Characterization

The microstructural morphology and elemental distribution of the SIAM sensing fibers were examined using a scanning electron microscope (SEM, Model E1856-C2B, USA) equipped with energy-dispersive X-ray spectroscopy (EDX). Functional groups were identified by Fourier transform infrared (FTIR) spectroscopy on a TENSOR-27 spectrometer (Germany). The crystalline structure was characterized via X-ray diffraction (XRD) using a D8 Advance diffractometer (Germany). Surface elemental composition and chemical states were investigated by X-ray photoelectron spectroscopy (XPS) on a Thermo Scientific K-Alpha instrument (USA). Specific surface area and pore size distribution were determined from nitrogen adsorption–desorption isotherms using the Brunauer–Emmett–Teller (BET) method on a Tristar II analyzer (USA). Tensile tests were performed on a universal testing machine (Instron 3365, USA) at a crosshead speed of 100 mm/min. Electrical resistance and output voltage of the fibers were measured with a digital multimeter (UT61E + , China). Thermal stability was evaluated by thermogravimetric analysis (TGA) on a Mettler Toledo instrument (USA), with samples heated from 30 to 800 °C at a rate of 10 °C min^−1^ under a nitrogen atmosphere.

## Results and Discussion

### Fabrication of CO-Temperature SIAM Bimodal Sensing Fiber

**Fig. 1:**
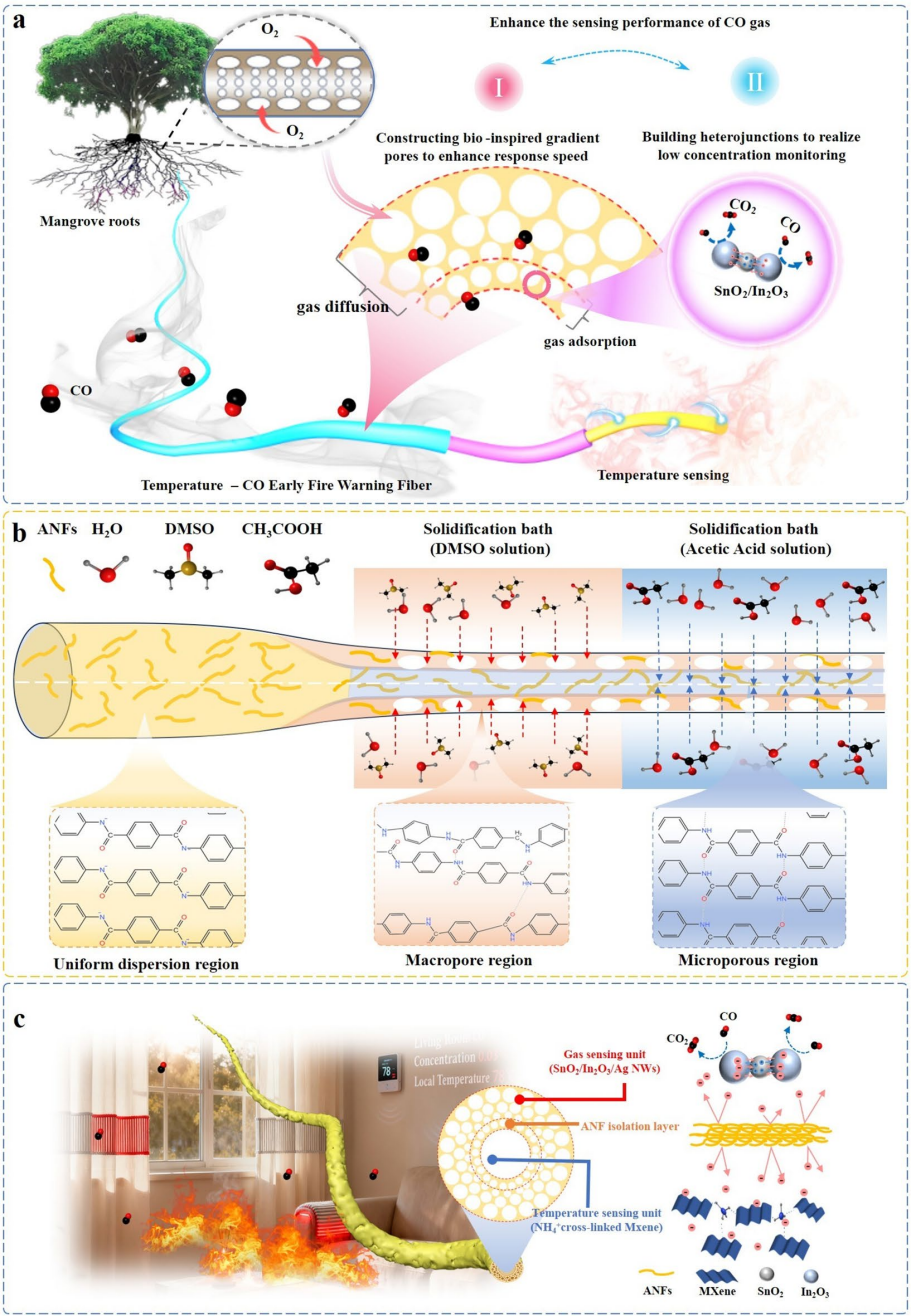
Concept description and fabrication of biomimetic gradient porous SIAM sensing fibers. **a** Preparation of SIAM sensing fibers and inspiration for gradient pore structure. **b** Gradient-induced phase separation technology for forming gradient pores sheath. **c** Application scenarios and cross-sectional structures of SIAM early fire-warning fibers

This work successfully constructed a dual-parameter SIAM fire warming fiber via a coaxial wet-spinning technology. The resulting SIAM sensing fiber comprises a CO sensing sheath made from a SnO_2/_In_2_O_3_ heterojunction/aramid nanofiber (ANF) composite with biomimetic gradient porosity, an ANF decoupling isolation layer, and a temperature sensing core composed of NH_4_^+^ crosslinked MXene, enabling integrated dual-mode sensing for CO and temperature (Fig. 1a). In this study, two-dimensional nanosheets Ti_3_C_2_T_X_ MXene were prepared by selectively etching the aluminum layer from Ti_3_AlC_2_ (Fig. S1) [43]. XRD analysis showed that the (002) diffraction peak of MXene shifted to the left, and the (104), (101), and (004) crystal planes completely disappeared after etching and ultrasonic treatment, indicating that Ti_3_AlC_2_ was successfully etched into a single-layer Ti_3_C_2_T_X_ MXene (Fig. S2). Subsequently, during wet spinning, the negatively charged MXene in the core spinning solution undergoes electrostatic crosslinking with NH_4_^+^ in the coagulation bath and forms a stable spatial network structure (Fig. S3). This enables the formation of a robust, conductive path in the MXene core layer, significantly improving carrier migration within the MXene and thereby enhancing the thermoelectric performance of the MXene core layer. In addition, SnO_2_/In_2_O_3_ gas-sensing material and Ag NWs were added to the sheath of SIAM sensing fibers (Fig. S4), forming an *n*–*n* heterojunction structure between n-type semiconductor In_2_O_3_ and SnO_2_. At the same time, Ag NWs served as a bridge connecting the SnO_2_/In_2_O_3_ gas-sensing material dispersed in ANFs.

As shown in Fig. 1a, a biomimetic gradient porous structural design is achieved, transitioning from large pores in the outer shell layer to micropores in the inner shell layer. This design is realized through gradient-induced phase separation technology, which regulates the proton exchange rate during the wet-spinning process [44, 45]. This structure facilitates the diffusion and adsorption of CO gas into the SIAM sensing fiber shell, thereby substantially improving the response speed for CO gas. The three-layer structure of SIAM sensing fibers is shown in Fig. S5. Figure 1b shows the proton exchange process of the SIAM sensing fiber shell during wet spinning. When the sheath ANF spinning solution is extruded from the nozzle, it first enters a 50% DMSO aqueous solution coagulation bath. DMSO was introduced into the coagulation bath to diminish the non-solvent strength of the coagulation bath, thereby significantly slowing the dual diffusion rates of non-solvent inward diffusion and solvent outward diffusion. As the driving force for phase separation diminishes, the explosive instantaneous separation evolves into a more gradual and mild phase separation process. During this slower phase separation, the rigid ANF molecular chains exhibit extended relaxation times, allowing for adjustment and assembly into a more stable phase separation structure. In addition, the polymer poor phase (non-solvent droplets) also has sufficient time to grow through the Ostwald ripening mechanism. Adjacent droplets merge and connect, small droplets dissolve, and large droplets grow, forming larger, more connected pores in the fiber’s outer layer sheath. At this point, the solidification front slowly advances from the fiber outer shell layer to the inner shell layer, but has not yet reached the interior. Afterward, the SIAM sensing fiber enters the acetic acid coagulation bath (10% CH_3_COOH aqueous solution) from the DMSO coagulation bath, and the environment undergoes drastic changes. A large number of acetic acid molecules and amide bonds (–NH–CO–) on the aramid molecular chain are combined through hydrogen bonding, promoting rapid protonation of amide nitrogen atoms and triggering a new round of faster phase separation. During this subsequent phase separation process, ANFs are accelerated to solidify, rapidly forming a rigid thr1ee-dimensional network. The aramid molecular chains lack time for large-scale adjustment and rearrangement and are quickly frozen in their current positions, ultimately forming dense, fine pore structures. By precisely controlling the fibers solidification in two steps, a gradient pore structure is formed in the shell layer of the SAM sensing fiber, gradually transitioning from large pores in the outer shell layer to small pores inside. The large pores (> 10 *μ*m) of the outer shell facilitate efficient diffusion of CO into the interior of the sensing fiber. Meanwhile, the micropores (< 3 *μ*m) of the inner shell provide a high specific surface area for the adsorption of CO onto SnO_2_/In_2_O_3_. This gradient pore structure significantly enhances the CO sensing performance and supports applications in early fire-warning systems (Fig. 1c).

### Microstructure of SIAM Sensing Fiber

**Fig. 2:**
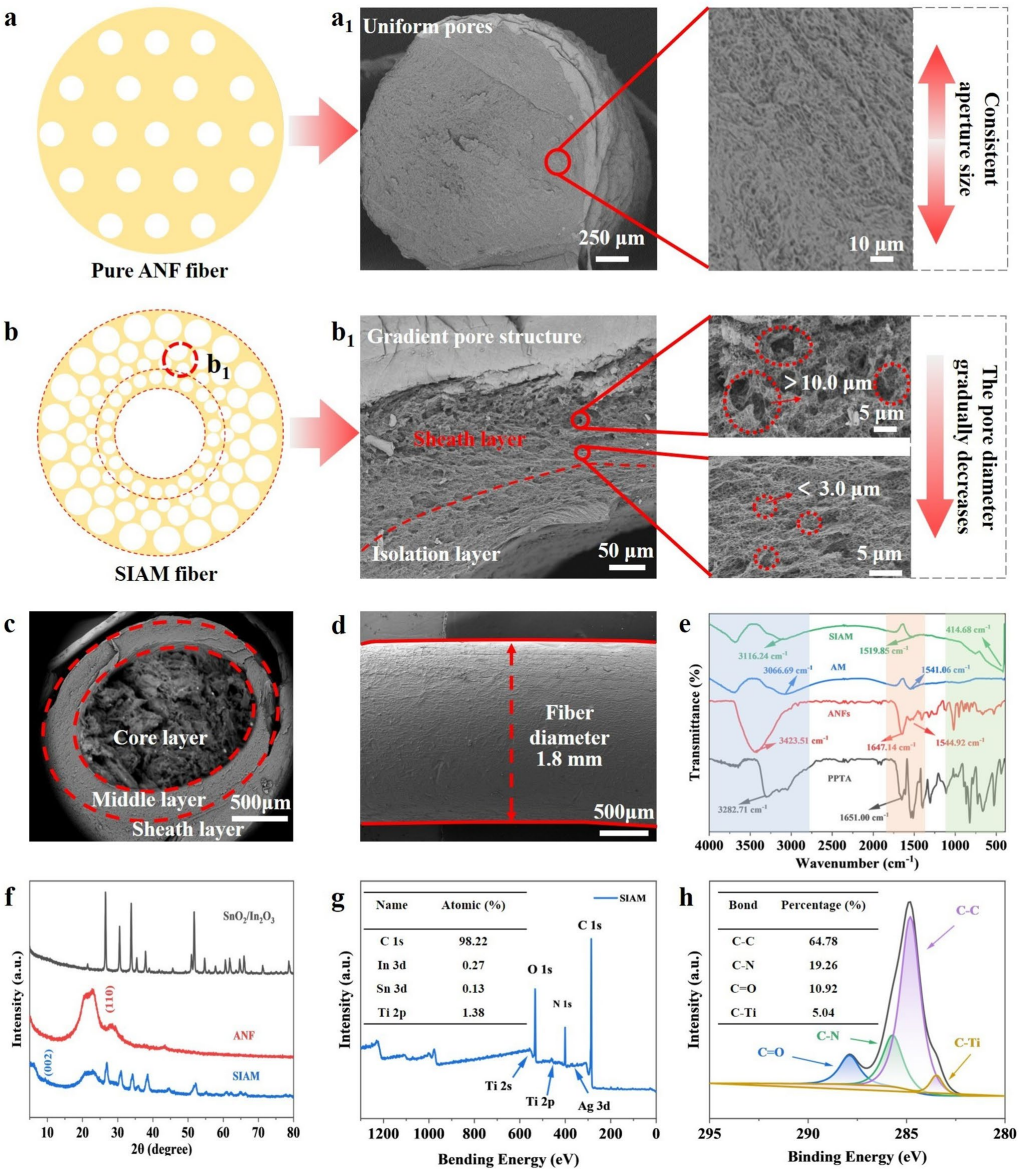
Microscopic characterization of SIAM sensing fibers. **a** Cross section of ANFs prepared by wet spinning using water as a coagulation bath. **b** Cross section of ANFs prepared by wet spinning using a two-step gradient solidification method. **c, d** Cross section and surface images of SIAM sensing fibers. **e** Infrared spectroscopy testing of PPTA fabrics, ANFs fibers, AM fibers, and SIAM fibers. **f** XRD patterns of SnO_2_/In_2_O_3_ gas-sensitive materials, ANF, and SIAM fibers. **g, h** XPS of SIAM fibers and precise spectral analysis of C elements

As shown in Fig. 2a, the cross section of the ANF aerogel fiber fabricated using pure water as the coagulation bath exhibits a uniformly dense small pore. This structure significantly hinders the movement or diffusion of gas molecules into the interior. In contrast, the cross section of SIAM sensing fibers prepared via gradient-induced phase separation technology clearly reveals that the outer shell layer contains larger-diameter pores, which transition inward to micropores in the inner shell layer (Fig. 2b). Figure 2c shows the complete and actual cross section of the SIAM sensing fiber, where the three-dimensional spatial network structure of the thermoelectric material MXene is clearly visible at the center. This observation indicates the successful crosslinking of MXene through NH_4_^+^ ions, which effectively prevents stacking and facilitates the formation of a stable conductive network. Figure 2d shows the smooth longitudinal surface of the SIAM dual-mode sensing fiber. EDX elemental testing revealed the element composition (Fig. S6) of the SIAM dual-mode sensing fiber, with elements such as C, Ti, Sn, and In. This finding corroborates the successful incorporation and uniform distribution of the SnO_2_/In_2_O_3_ gas-sensing material. Furthermore, the uniform distribution of the Ti element, along with the cross-sectional electron microscopy image of the SIAM sensing fiber, confirms the successful construction of the conductive network within sensing core.

Further FTIR spectroscopic analysis was conducted to analyze the chemical structure of SIAM sensing fibers and verify the hydrogen bonding interactions among substances (Figs. 2e and S7). The results showed that ANF exhibited characteristic peaks at 3423.51 and 1544.92 cm^−1^, corresponding to the N–H peak and amide characteristic peak, respectively. Compared with PPTA, the N–H characteristic peak in the FTIR spectrum of ANF shows a slight blue shift, which may result from the disruption of numerous intermolecular hydrogen bonds during the preparation process of ANF. Although hydrogen bonds can be restored through reprotonation, the nanoscale hydrogen bond network remains disordered and weakened, contributing to the observed blue shift of the N–H peak. Concurrently, the FTIR spectrum of the SIAM sensing fiber revealed the metal lattice vibration peak of SnO_2_ at 615 cm^−1^ and that of In_2_O_3_ at 540 cm^−1^. In addition, from the FTIR spectral results, the characteristic peak corresponding to the stretching vibration of C=O in ANF shifted from 1647.14 to 1519.85 cm^−1^ in the SIAM sensing fiber, showing a slight red shift phenomenon, which can be attributed to the formation of hydrogen bonds between ANF and MXene, enhancing the interaction force between fiber layers. In addition, as evidenced by the XRD spectrum (Fig. 2f), the SIAM dual-mode sensing fiber exhibited characteristic peaks at 23.63° and 5.80°, corresponding to the (110) plane of ANFs-AF and the (002) plane of MXene, respectively. Characteristic peaks of SnO_2_ at 34.00° and In_2_O_3_ at 31.00° were also observed in the SIAM sensing fiber, confirming the successful loading of SnO_2_/In_2_O_3_ gas-sensing material onto the SIAM sensing fibers. Due to the low content of Ag NWs, only weak characteristic peaks of Ag NWs are observed in the XRD pattern of SIAM sensing fibers (Fig. S8).

Subsequently, further analysis of the chemical structure within the SIAM sensing fiber was conducted using XPS analysis, with the spectra illustrated in Fig. 2g, h. All spectra were referenced to the C 1*s* peak at a binding energy of 284.8 eV. The results indicate that the SIAM dual-mode sensing fiber prepared via wet spinning exhibits characteristic peaks of varying intensities (C 1*s*, Ti 2*p*, Sn 3*d*, and In 3*d*) across a broad XPS spectral range. The characteristic peaks in the C 1*s* spectrum include C=O (287.9 eV), C–N (285.7 eV), C–C (284.8 eV), and C–Ti (283.5 eV). Meanwhile, the high-resolution Ti 2*p* spectrum of the SIAM sensing fiber reveals the presence of Ti–O (Ti 2*p*_1/2_) at 464.5 eV, Ti–C (Ti 2*p*_1/2_) at 461.5 eV, TiO_2_ at 459.0 eV, Ti–O–C at 455.8 eV, and Ti–C bonds at 455.0 eV (Fig. S9). In summary, these results indicate that strong bonding energy and interaction have formed between ANF and MXene, which contribute to the formation of a continuous and uniform fiber structure. Combined with SEM images of the SIAM sensing fiber cross section and the uniformly distributed Ti element observed in EDX elemental mapping, this confirms that the MXene network in the core layer of the SIAM sensing fiber forms a continuous, stable conductive pathway.

### Flame Retardancy and Thermal Stability Property of SIAM Sensing Fiber

**Fig. 3:**
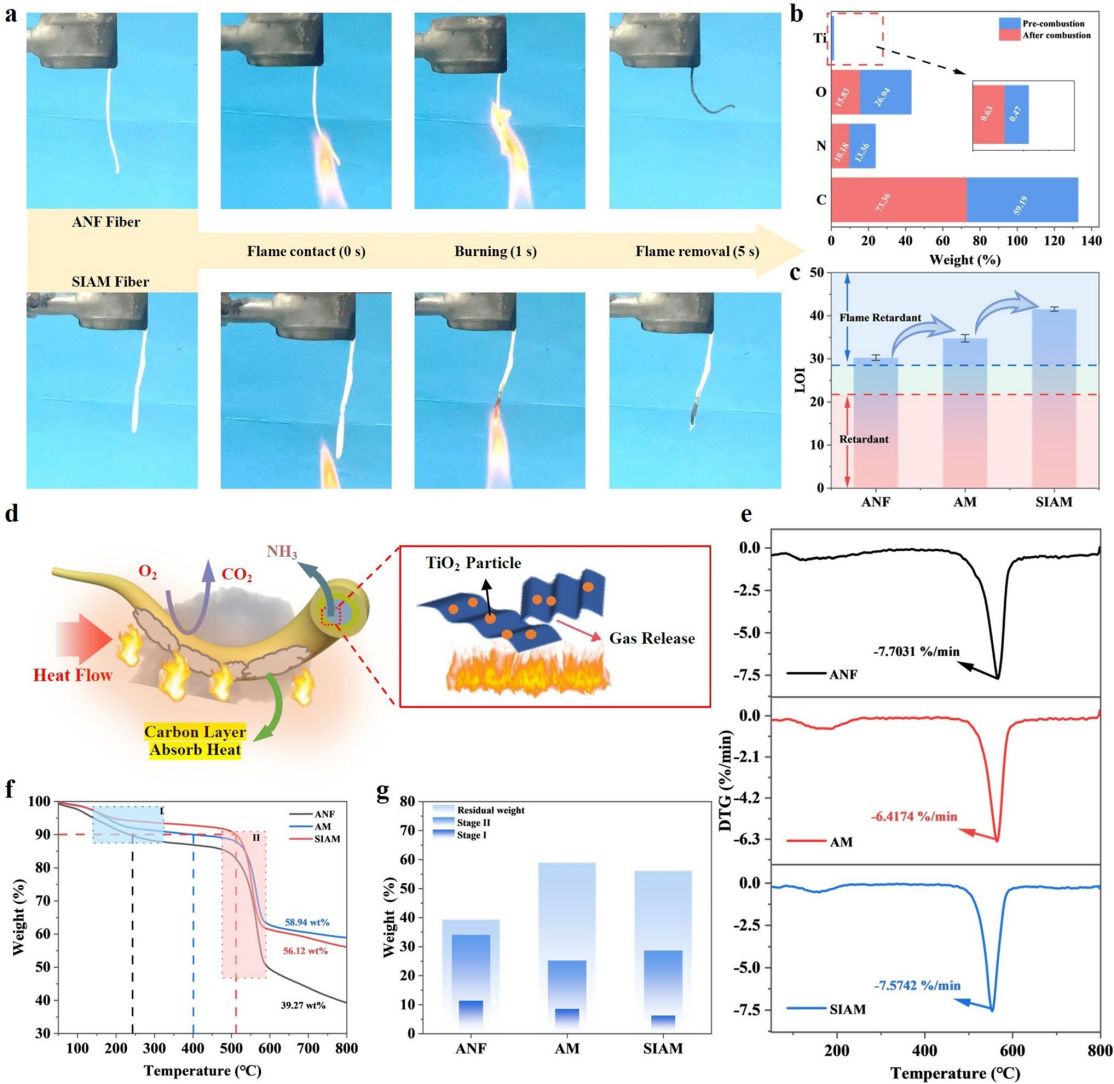
Flame retardant properties of SIAM sensing fibers. **a** Comparison experiment of flame burning between ANF fiber and SIAM sensing fiber. **b** Elemental analysis of SIAM sensing fibers before and after combustion. **c** LOI of ANF fiber and SIAM sensing fiber. **d** Schematic diagram of flame retardant mechanism of SIAM sensing fiber. **e–g** Thermogravimetric testing of AMF, AM, and SIAM sensing fibers

A flame contact test was conducted on the SIAM sensing fiber to investigate its flame retardant properties (Fig. 3a). Compared to the pure ANF fiber, the SIAM sensing fiber exhibited minimal combustion when exposed to the alcohol lamp flame. Furthermore, it self-extinguished immediately upon removal from the flame, without causing significant damage to the fiber structure (Movie S1). In contrast, the ANF fiber showed significant combustion upon contact with the alcohol lamp flame, resulting in substantial structural damage. Subsequently, the flame contact test results for SIAM and ANF fibers were further analyzed (Fig. S10). After burning for 7*s*, it was found that the residual length ratio of SIAM sensing fibers was as high as 92.05%, which was 38.09% higher than that of pure ANF fibers, confirming the excellent stability performance of SIAM sensing fibers. Subsequently, the elemental content of the SIAM sensing fiber before and after combustion was analyzed, as shown in Fig. 3b. After combustion, the contents of C and Ti elements in the SIAM sensing fiber increased by 14.17% and 0.16%, respectively, while the contents of O and N elements decreased by 11.11% and 3.18%, respectively. This phenomenon is likely due to the combustion behavior of the ANF, which serves as the skeleton of the SIAM sensing fiber. Its combustion influences the variation in elemental content within the SIAM sensing fiber. The molecular chains of aramid primarily consist of aromatic rings and amide bonds (–NH–CO–). Under the attack of a high-temperature flame, the amide bonds in the macromolecular chains of the aramid nanofiber, which are the weakest link, break first, releasing gaseous combustion products (such as carbon monoxide, carbon dioxide, and nitrogen-containing gases). Consequently, O and N elements are dissipated primarily along with the breakage of the amide bonds in the aramid macromolecular chains, leading to a decrease in their content. In comparison, the contents of C and Ti elements (from MXene) increase relatively due to their higher stability.

Limiting oxygen index (LOI) testing was conducted to characterize the flame retardancy of SIAM sensing fibers (Fig. 3c). Compared to pure ANF and AM fibers (including MXene), the LOI value of SIAM sensing fibers was higher, confirming their superior flame retardancy over ANF and AM fibers. To better understand the flame retardancy of the SIAM sensing fiber, its flame retardant mechanism was simulated and analyzed, as schematically illustrated in Fig. 3d. The combustion of the SIAM sensing fiber involves complex physical and chemical changes, which can be divided primarily into gas-phase and solid-phase inhibition. In the gas phase, the ANF matrix releases non-combustible gases such as CO_2_, NO_2_, and H_2_O during combustion. These gases dilute the surrounding oxygen and flammable gas concentrations, thereby inhibiting flame propagation. Additionally, the NH_4_^+^-crosslinked MXene in the core layer can release NH_3_ upon heating, further contributing to gas-phase flame retardancy. Regarding solid-phase inhibition, the aramid nanofiber undergoes carbonization during combustion, forming a dense and stable continuous carbon layer that isolates oxygen and heat, preventing further flame spread. Furthermore, the Ti element in the core layer of the MXene forms TiO_2_ nanoparticles at high temperatures. These TiO_2_ nanoparticles possess extremely high thermal stability (melting point of 1800 °C) and adhere to the surface of the unburned matrix, forming a rigid physical barrier. In addition, TiO_2_ acts as a Lewis acid, catalyzing carbonization in polymers and promoting crosslinking and aromatization reactions during the pyrolysis of polymer molecules, rather than directly cracking into flammable small molecules. Through Raman testing of ANF fibers and SIAM sensing fibers after combustion, it was found that the *I*_*D*_/*I*_*G*_ of ANF fibers was 1.0643, indicating that the carbon layer generated by ANF fibers after combustion has a low degree of graphitization and is composed of a large number of small graphite grains and amorphous carbon regions. The I_D_/I_G_ of SIAM sensing fiber after combustion is 1.0264, which is lower than the *I*_*D*_/*I*_*G*_ value of ANF fiber. From the above results, it can be seen that the catalytic effect of TiO_2_ helps to form a denser, more stable, and graphitized carbon layer (Fig. S11), thereby providing more effective protection for unburned fibers. To further investigate the thermal stability, thermogravimetric tests were conducted on ANF fiber, AM fiber, and the SIAM sensing fiber (Fig. 3e-g). The results indicated that the residual mass of the ANF fiber was 39.27 wt%, while the residual masses of the AM fiber and SIAM sensing fiber were 58.94 wt% and 56.12 wt%, respectively, both higher than that of the pure ANF fiber. Moreover, the maximum mass loss rate of the SIAM sensing fiber was 7.5742% min^−1^, which is lower than that of the ANF fiber (7.7031% min^−1^). Figure 3f shows that these three fibers undergo similar thermal decomposition processes, both consisting of two main stages. The first stage, occurring around 100 °C, primarily involves the evaporation of internal moisture and the decomposition of hydrophilic groups; the SIAM sensing fiber undergoes a mass loss of about 6.22% at this stage. The second stage occurs between 500 and600 °C, where the macromolecular chains of the aramid nanofibers begin to decompose and the main fiber structure starts to degrade; the mass loss of the SIAM sensing fiber at this stage is approximately 28.63%. Based on the analysis of the above results, the SIAM sensing fiber exhibits better thermal stability compared to the ANF fiber and AM fiber, thereby enabling reliable sensing ability in early fire-warning processes.

### Pore Structure and Mechanical Properties Analysis of SIAM Sensing Fiber

**Fig. 4:**
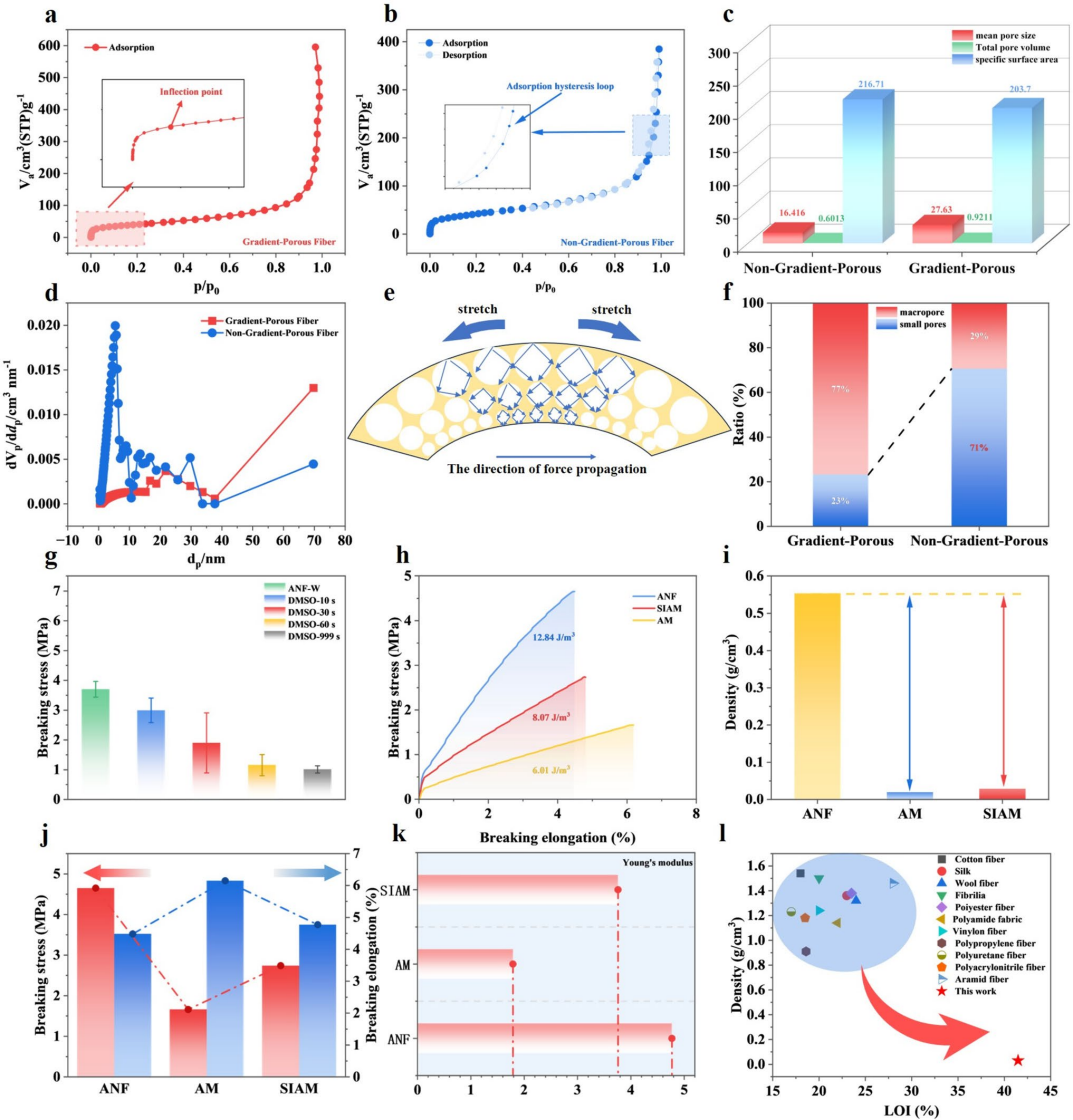
Mechanical properties of SIAM sensing fiber. **a, b** Adsorption isotherms of gradient porous fiber and non-gradient porous fiber under N_2_. **c** Comparison of average pore size, pore volume, and specific surface area between gradient porous fiber and non-gradient porous fibers. **d** Pore size distribution of the SIAM fiber. **e** Stress distribution within the SIAM sensing fiber under external force. **f** Respective proportions of small and large pores in SIAM sensing fiber. **g** Mechanical properties of SIAM fibers soaked in DMSO coagulation bath for different time. **h** Stress–strain curves, **i** density comparison, **j** fracture stress and elongation at break comparison, and **k** Young's modulus comparison of ANF fiber, AM fiber, and SIAM fiber. **l** Comparison of density and LOI value of SIAM sensing fibers with commercial fibers

The BET test results of SIAM sensor fibers prepared by gradient-induced phase separation technology and SIAM sensor fibers solidified in a water bath are shown in Fig. 4a and b. The adsorption isotherm of the SIAM gradient porous fiber exhibits an inflection point near monolayer coverage, characteristic of a Type II isotherm, indicating the presence of large pores within the fiber structure. In contrast, the adsorption isotherm of the fiber without gradient porous shows a distinct hysteresis loop, consistent with a Type IV isotherm, which reflects its internal mesoporous structure. Further analysis of the BET data provides the average pore size, pore volume, and specific surface area for both fibers (Fig. 4c). Comparative analysis reveals that the SIAM gradient porous fiber possesses a larger average pore size of 27.63 nm compared to 16.416 nm for the non-gradient-porous fiber. Conversely, the non-gradient porous fiber exhibits a greater specific surface area (216.71 m^2^ g^−1^) than the gradient porous fiber (203.7 m^2^ g^−1^). These results suggest that the non-gradient porous fiber contains numerous small pores, whereas the gradient porous SIAM fiber exhibits a significant volume of both large and small pores. This confirms the successful fabrication of SIAM sensing fibers with macropores using the gradient-induced phase separation technology. By comparing the pore size distribution of gradient porous fibers with that of non-gradient porous fibers (Fig. 4d, f), it was observed that, within the range of 0–40 nm, small pores in non-gradient porous fibers constituted 71% of the total pore volume, significantly higher than the 23% observed in gradient porous fibers. Conversely, for pore sizes exceeding 40 nm, large pores in gradient porous fibers represented 77% of the total pore volume, substantially surpassing the 29% proportion of large pores in non-gradient porous fibers. These findings indicate that a large number of macropores were successfully introduced into the SIAM sensing fibers and formed a gradient porous sheath from outer (> 10 *μ*m) to inner (< 3 *μ*m) regions via gradient-induced phase separation technology.

During the wet spinning of SIAM sensing fibers, by regulating the proton exchange rate in the coagulation bath, pores of different sizes are generated in the outer and inner shell layers of the fibers. Therefore, the duration of the fiber in the coagulation bath determines the ratio between the large pores in the outer shell and the small pores in the inner shell of the fiber, and this ratio significantly affects the mechanical properties of the fiber. The presence of macropores introduces more defects within the fiber structure. Under applied stress (Fig. 4e), significant stress concentration occurs at the edges of these pores, particularly those with irregular shapes, facilitating the initiation and rapid propagation of microcracks, ultimately leading to fiber fracture. In contrast, fibers containing uniformly distributed small pores exhibit an increased effective load-bearing cross-sectional area, allowing stress to be distributed more homogeneously throughout the fiber. Even if microcracks initiate at pore edges, they require more energy to propagate into adjacent pores. Therefore, under the same conditions, the strength of fibers with small pores is higher than that of fibers with large pores. In the experiment, the immersion time of fibers in the DMSO coagulation bath was optimized. After experiments, fibers immersed in the DMSO coagulation bath for 10 s (DMSO-10 s), 30 s (DMSO-30 s), and 60 s (DMSO-60 s), and fibers continuously immersed in the DMSO coagulation bath without undergoing a second coagulation bath treatment were obtained and named DMSO-999 s. For comparison, ANF controls were fabricated using conventional wet-spinning with water as the sole coagulation bath, designated ANF-W. The stress–strain tests were conducted on these five types of fibers, and the results are shown in Fig. 4g. As the immersion time of the fibers in the DMSO coagulation bath increased, the fibers’ strength decreased accordingly. The longer the immersion time of fibers in DMSO coagulation bath, the higher the proportion of macropores in the fibers, and the poorer the strength of the fibers. In order to balance the mechanical properties and gradient pore properties required for fibers in daily use, the DMSO coagulation bath immersion for 30 s was selected as the subsequent experimental method (unless otherwise specified, SIAM sensing fibers were prepared using DMSO coagulation bath immersion for 30 s in subsequent experiments). Subsequently, the mechanical properties and densities of ANF fiber, AM fiber, and SIAM sensing fiber were compared. The results are shown in Fig. 4h–k. As the intermediate layer of SIAM is made of pure aramid as an isolation layer, it firmly locks the overall structure of the fiber and improves the mechanical properties of the SIAM sensing fiber. The fracture process of SIAM sensing fiber is shown in Fig. S12. Therefore, the mechanical strength and Young’s modulus of the SIAM sensing fibers are higher than those of AM fibers, which are also core–sheath fibers, but slightly lower than those of ANFs fibers. Moreover, due to the MXene spatial network structure and gradient pores in the core layer, the density of SIAM sensing fibers is significantly reduced. Furthermore, the LOI value and density of SIAM sensing fibers were compared with those of commonly used fibers in the market (Fig. 4l), demonstrating excellent flame retardancy and lightweight properties.

### Temperature Sensing Performance of SIAM Sensing Fiber

**Fig. 5:**
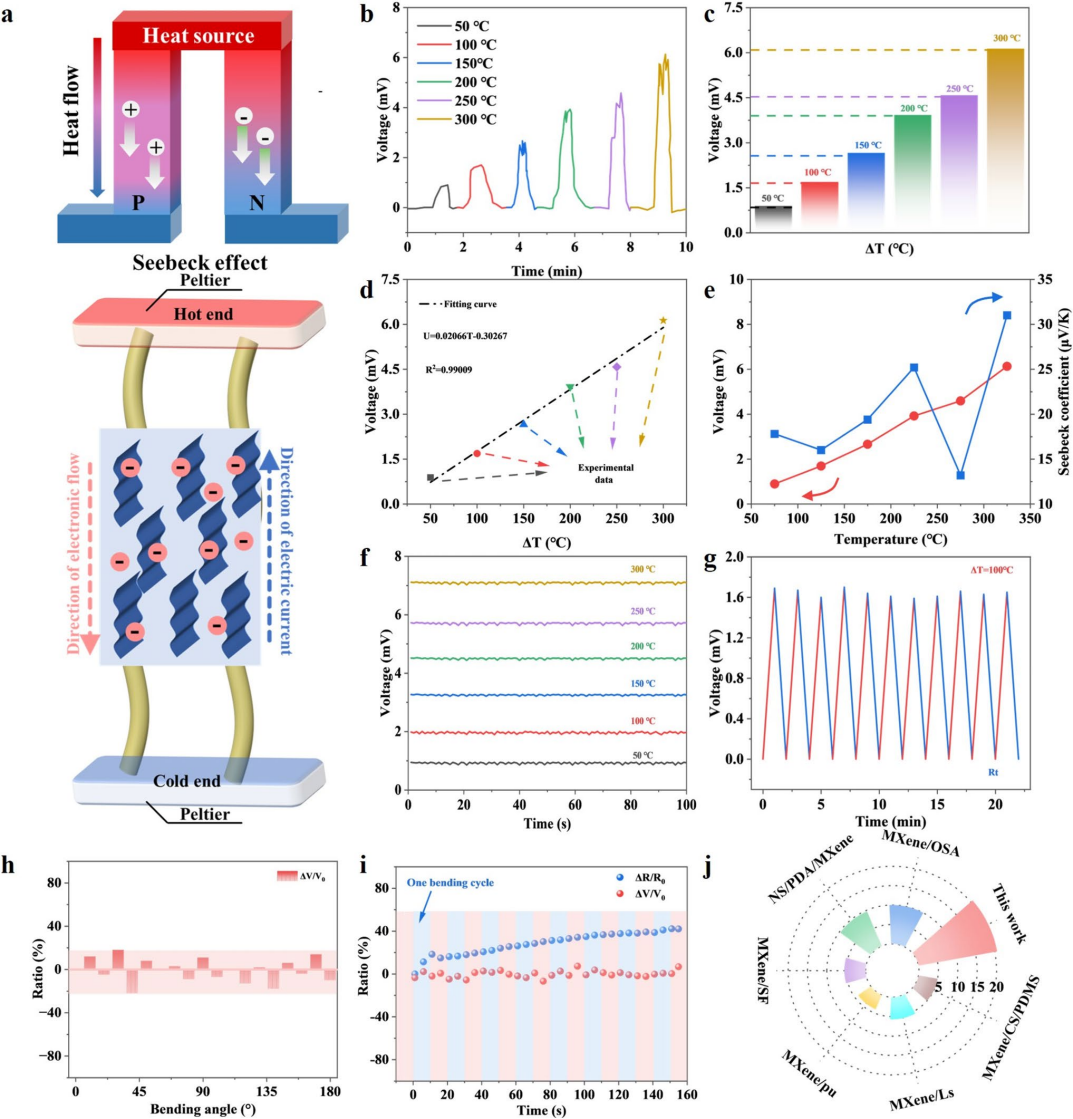
Temperature sensing performance of SIAM sensing fiber. **a** Schematic diagram of Seebeck effect and temperature sensing mechanism of SIAM sensing fiber. **b, c** Relationship between the output voltage and temperature difference of the SIAM sensing fiber. **d** Linear regression equation between temperature difference and output voltage. **e** Seebeck coefficient of MXene in SIAM sensing fibers at different temperatures. **f** Long-term voltage output of SIAM sensing fibers at different temperatures. **g** Voltage output of SIAM sensing fibers under multiple heating and cooling cycles [46]. **h** Output voltage of SIAM sensing fibers under different bending angle. **i** Voltage output and resistance changes of SIAM sensing fibers under long-term bending (180°) and recovery (0°) cycles. **j** Seebeck coefficient of MXene in SIAM sensing fibers with that of MXene-based temperature sensors in other works

The thermoelectric properties of SIAM sensing fibers were experimentally tested. Five SIAM sensing fibers are neatly arranged, with a length of approximately 6 cm. The two ends of the fibers are placed on a Peltier element (one end for heating and the other end for cooling, with a distance of 5 cm between the hot and cold ends). The change in output voltage across the fiber core was measured by altering the temperature at both ends of the fiber. The temperature sensing mechanism is shown in Fig. 5a, which is primarily attributed to the temperature difference causing the charge carriers (electrons in the case of MXene) within MXene to move in a directional flow, transferring from the high-temperature region to the low-temperature region. Over time, carriers accumulate in the low-temperature region, eventually forming a region of high electrical potential. When connected to an external circuit, a significant potential difference develops across the low-temperature and high-temperature regions. Figure 5b, c shows the voltage changes across the SIAM sensing fiber within a temperature difference range of 50–300 °C. It is evident that as the temperature increases, the voltage across the SIAM sensing fiber correspondingly increases. Subsequent detailed analysis of the data revealed a good linear relationship between the voltage across the SIAM sensing fiber and the temperature difference (Fig. 5d). The calculated linear regression equation is “*U* = *0.02066 T–0.30267”* (where *U* is the voltage across the fiber ends, and *T* is the temperature difference between the fiber ends), with a linear fit (*R*^2^) of 99.01%. Therefore, based on the above calculation, measuring the output voltage across the SIAM sensing fiber allows it to be substituted into the aforementioned linear regression equation to calculate the temperature difference between the fiber ends, achieving the mutual conversion of electrical and temperature signals. Furthermore, the Seebeck coefficient of NH_4_^+^ cross-linked MXene in the SIAM sensing fiber was measured at different temperature points (Fig. 5e). The results show that MXene maintains a relatively good Seebeck coefficient within the 50–300 °C range, indicating excellent efficiency in converting thermal energy to electrical energy. Considering the influence of fiber length (hot end/cold end distance) on its sensing performance, SIAM sensing fibers with different cold and hot end distances were set up for temperature sensing testing in this study. The test results indicate that the output voltage difference of SIAM sensing fibers with cold and hot end distances of 8 cm and 5 cm at 100 °C is very small, with average values of 1.90 and 1.70 mV, respectively (Fig. S13). Both are close to the calculated theoretical value of 1.76 mV at this temperature. The reason for the difference between the two may be due to the mutual influence of heat generated by the cold and hot ends of the fiber as the distance between the two ends decreases, resulting in a shorter actual temperature difference on the fiber. The output voltage of the SIAM sensing fiber with a distance of 2 cm between the hot and cold ends is about 0 mV, indicating that at this distance, the temperature difference on the fiber is no longer significant and cannot generate an output voltage signal.

Subsequently, the output voltage across the SIAM sensing fiber was measured under different temperature differences (Fig. 5f). The test results indicate that once the temperature difference stabilizes, the output voltage of the SIAM sensing fiber remains stable over an extended period, thereby demonstrating the long-term stability of its voltage output. In addition, to evaluate the repeatability of the temperature sensing function, a cyclic test was conducted, in which one end of the same batch of SIAM sensing fibers was heated to a temperature difference of 100 °C and then cooled to room temperature. Each cycle lasts for 2 min, including heating and natural cooling, and is repeated for a total of 11 cycles. The results indicate that the output voltage distribution of SIAM sensing fiber is around the theoretical value of 1.76 mV. During the continuous cycle, the maximum fluctuation amplitude of the output voltage was less than 0.1 mV, and no significant baseline drift or performance degradation was observed. To evaluate the accuracy of the dataset (Fig. 5g), the root mean square error (RMSE) between the measured values and the theoretical values was calculated. The calculated RMSE is 0.124, indicating that the slight difference between the measured value and the true theoretical value is within an acceptable range. This error mainly comes from environmental temperature fluctuations, changes in contact resistance during testing, and inherent noise in the data acquisition system. However, overall, the sensor exhibits good stability and reliable repetitive output capability. Therefore, the above analysis and calculations indicate that the temperature sensing performance of SIAM sensing fiber has high stability and repeatability. Considering that the SIAM sensing fiber may undergo bending or twisting for various reasons in practical applications, the output voltage was characterized under different bending angles (Fig. 5h). The results indicate that under bending conditions, the fluctuation in output voltage is minimal, with a calculated standard deviation of 0.11, demonstrating that the data is relatively concentrated. In addition, the effect of repeated bending on temperature sensing performance was evaluated under cyclic bending recovery conditions (Fig. 5i). During the testing process, the SIAM sensing fiber was repeatedly bent to 180° under a temperature difference of 100 ℃ and then restored to 0° (bending cycle was performed every 10 s). Each bending cycle stage is marked in Fig. 5i to clearly display the dynamic signal changes during the deformation process. The results indicate that the output voltage variation of SIAM sensing fiber remains within 9%, while the resistance fluctuation gradually stabilizes within the range of 30–40% during repeated bending cycles. These results indicate that the conductive network of fibers is to some extent affected by mechanical deformation. Nevertheless, SIAM sensing fibers maintain continuous conductivity and effective temperature sensing function during the bending process. In addition, during repeated deformation cycles, the voltage response curve exhibits relatively good reproducibility. This behavior can be attributed to the cross-linking effect of NH_4_^+^ on MXene nanosheets and the hydrogen bonding interaction between MXene and ANF, which helps maintain the integrity of the conductive pathway under deformation. In addition, we also conducted stability tests on the output voltage of SIAM sensing fibers after multiple bending cycles. According to the test results (Fig. S14), after 200 cycles of bending, the output voltage of SIAM sensing fiber at a temperature difference of 100 °C is between 1.5 and 1.7 mV, with an error of less than 15% compared to the calculated theoretical value of 1.76 mV. This indicates that it has a certain degree of bending stability and can maintain good temperature monitoring ability even after multiple bends. Therefore, SIAM sensing fibers have certain mechanical adaptability and potential for flexible sensing applications. The Seebeck coefficient of MXene in the SIAM sensing fiber was measured in this study (*S* = *ΔV/T* = 20.6 *μ*V K^−1^, where* S* is the Seebeck coefficient, *ΔV* is the output voltage, and* T* is the temperature difference). When compared to the Seebeck coefficients of MXene-based temperature sensors reported in other studies, the SIAM sensing fiber developed in this work demonstrates higher temperature sensing capabilities (Fig. 5j). This enhanced performance can be attributed to the cross-linking effect of NH_4_^+^, which facilitates the construction of continuous and stable carrier migration channels between MXene, significantly improving its thermoelectric effect.

### CO Sensing Performance of SIAM Sensing Fiber

**Fig. 6:**
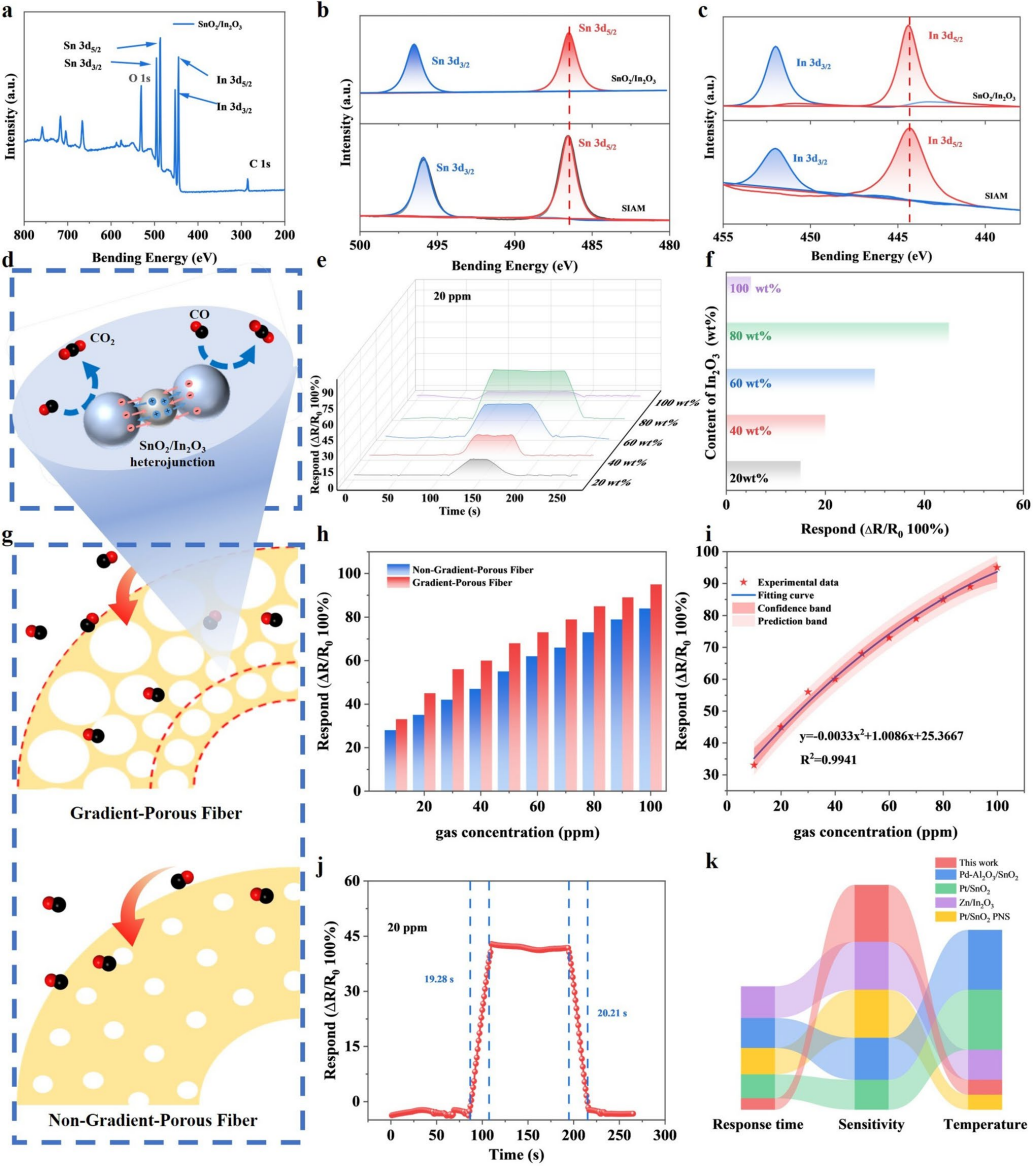
CO sensing performance of SIAM sensing fibers. **a** XPS wide-spectrum analysis of SnO_2_/In_2_O_3_ heterojunction. **b, c** Sn 3*d* and In 3*d* of SIAM fibers and SnO_2_/In_2_O_3_ heterojunction. **d** Reaction process of SnO_2_/In_2_O_3_ heterojunction with CO gas. **e, f** Effect of mass percentages of In_2_O_3_ in SnO_2_/In_2_O_3_ heterojunction on the CO gas-sensing performance in SIAM fibers. **g** Influence of gradient pores and non-gradient pores on CO gas-sensing performance in SIAM fibers. **h, i** Response of SIAM fibers to different concentrations of CO gas and the fitting curve. **j** Response curve of SIAM fibers to 10 ppm CO. **k** CO sensing response of SIAM fibers compared with those reported in the literature

When SnO_2_ and In_2_O_3_ come into contact to form a heterojunction, due to the Fermi level difference between the two, electrons will spontaneously migrate from the higher Fermi level In_2_O_3_ to SnO_2_ until the interface reaches a new Fermi level equilibrium. During this process, a significant built-in electric field was formed at the heterojunction interface, accompanied by the occurrence of band bending phenomenon [47, 48]. At the same time, the redistribution of electrons in the interface region causes a change in the electron concentration on the SnO_2_ side, resulting in the formation of a wider electron depletion layer at the heterojunction interface. The formation of this depletion layer will significantly increase the sensitivity of the material interface to surface-adsorbed oxygen and CO gas reactions. Figure 6a shows the full-scale XPS spectrum of SnO_2_/In_2_O_3_, confirming the presence of Sn, In, O, and C elements, with no peaks from other impurity elements detected. The presence of the C element can be attributed to adventitious carbon-based contaminants, such as adsorbed carbon-containing gases. Figure 6b, c presents comparative analyses of the high-resolution Sn 3*d* and In 3*d* spectra for the SnO_2_/In_2_O_3_ composite material and the SIAM sensing fiber, respectively. The spectra indicate that incorporating the SnO_2_/In_2_O_3_ composite into the aramid nanofiber matrix did not alter its intrinsic chemical structure, suggesting that no new chemical bonds formed in the SIAM sensing fiber that would affect its inherent sensing properties. Subsequently, CO gas-sensing experiments were conducted on the SIAM sensing fiber. SIAM sensing fibers were placed in a sealed gas chamber, with wires connected to both ends and linked to a multimeter to monitor real-time resistance changes. In this work, the response value of SIAM sensing fiber is defined as ΔR/R_0_ 100%, where R_0_ represents the baseline resistance of the sensor in air or initial state, and ΔR is the change in resistance of the sensing fiber after exposure to the target gas stimulus, which is the baseline resistance of the sensing fiber in air or initial state minus the real-time resistance of the sensing fiber after exposure to the target gas stimulus. The reaction process between the SIAM sensing fiber and CO gas is illustrated in Fig. 6d. Initially, O_2_ molecules adsorb onto the surface of the SnO_2_/In_2_O_3_ composite and capture electrons, forming chemisorbed oxygen species (O^2−^, O^−^, O_2_^−^). As electrons are captured, an electron depletion layer forms at the surface of the SnO_2_/In_2_O_3_ composite, leading to a significant increase in material resistance that gradually stabilizes in air. Upon exposure to CO gas, CO molecules react with the surface-adsorbed O_2_^−^ species to form CO_2_ and release electrons. This process leads to a reduction in the resistance of the SnO_2_/In_2_O_3_ composite material, thereby decreasing the resistance of the SIAM sensing fiber. Then comes the gas desorption stage, where after introducing air to reduce the concentration of CO gas, the generated CO_2_ gradually leaves the material surface and diffuses outside the fibers. The oxygen molecules in the air re adsorb onto the surface of the material and carry away electrons, resulting in an overall increase in the resistance of the fiber. Subsequently, the effect of In_2_O_3_ content on the CO sensing performance of the SnO_2_/In_2_O_3_ composite was investigated. As shown in Fig. 6e, f, the incorporation of In_2_O_3_ effectively enhances the sensing response of SnO_2_ toward CO gas. The optimal response was observed at 80 wt% In_2_O_3_, and thus, this composition was adopted for the SIAM sensing fiber in subsequent tests. This SnO_2_/In_2_O_3_ *n*–*n* heterojunction interface establishes an additional potential barrier to electron transport, leading to a high initial resistance in air for the SnO_2_/In_2_O_3_ composite. However, further increasing the In_2_O_3_ content led to a decline in the CO sensing response. This phenomenon can be attributed to the fixed total mass of the SnO_2_/In_2_O_3_ composite in the fiber. As the In_2_O_3_ content increases, the SnO_2_ content decreases proportionally, reducing the number of CO adsorption sites on SnO_2_ and consequently weakening the sensing response.

Furthermore, Fig. 6g shows a schematic diagram of the CO gas-sensing process of SIAM fibers with gradient pore sheath layer and control group without uniform pore sheath layer. It can be seen that the larger pores on the surface of the gradient pore sheath layer are more conducive to the diffusion of CO gas molecules inward, indicating that this structure promotes the CO sensing ability of SIAM fibers with gradient pore sheath. Figure 6h, i compares the CO sensing responses of SIAM fibers with gradient porous structures and those with non-gradient porous structures. It should be pointed out that the target application scenario of this study is early fire warning, with a focus on achieving high-sensitivity detection of trace amounts of CO gas. In the initial stage of a fire, the concentration of CO is usually within a few tens of ppm, so we focus on this low concentration range for system testing to meet the requirements for low concentration response accuracy and resolution in actual early warning. The results clearly demonstrate that the gradient porous structure significantly improves the CO response of the SIAM sensing fiber, highlighting the beneficial role of structural design in enhancing gas-sensing performance. The gradient pore structure is composed of large pores in the outer shell and small pores in the inner shell. The large pores in the outer shell facilitate the rapid entry of a large number of CO gas molecules into the SIAM sensing fiber. In contrast, the small pores in the inner shell provide a large specific surface area for CO gas adsorption, enabling reactions with the SnO_2_/In_2_O_3_ composite material within the sensing fiber and thereby improving sensing performance by approximately 15%. For non-gradient porous fiber, although its large number of small pores provides a higher specific surface area, the presence of small pores on the outer surface limits the rapid entry of CO gas into the fiber interior, resulting in a slower and weaker sensing response. Figure 6i presents the fitted response curve of the gradient porous SIAM sensing fiber toward CO gas, which follows the equation: *y* = *0.0033x*^*2*^ + *1.0086x* + *25.3667*, with correlation coefficient of 0.9941. These results indicate that the CO concentration in the environment can be accurately determined by measuring the resistance variation of the sensing fiber. Furthermore, Fig. 6j shows the response time and recovery time of SIAM fibers with gradient pores to 10 ppm CO gas. In addition, after testing, under the same testing conditions (same testing temperature and humidity, tested with different gases at a gas concentration of 100 ppm), the SIAM sensing fiber has the highest selectivity for CO gas (Fig. S15), and the resistance change caused by other gases is the smallest. It should be noted that the selective experiments in this study mainly selected ammonia and alcohol gases as interference gases to verify the basic recognition ability of the sensing fiber for CO gas. However, in actual fire or smoldering processes, the gas composition in the environment is often more complex. In addition to CO, there may also be multiple components such as CO_2_, water vapor, NO_x_, and smoke particles present simultaneously, all of which may have a certain impact on sensing performance. Therefore, the current selective testing results mainly focus on verifying the CO response characteristics of SIAM sensing fibers, and further research is needed on the sensing behavior under complex fire smoke conditions. Subsequently, relevant tests will be conducted in conjunction with a mixed atmosphere that is closer to the real fire environment to further evaluate the application performance of the sensing fiber in actual fire warning. As summarized in Fig. 6k, the gradient porous SIAM sensing fiber exhibits superior response speed and sensitivity to CO gas at room temperature compared to other CO sensors reported in the literature (Pt/SnO_2_ PNS [37], Zn/In_2_O_3_ [49], Pd/SnO_2_ [50], Pd-Al_2_O_3_/SnO_2_ [51]).

### Extended Application of SIAM Sensing Fiber

**Fig. 7:**
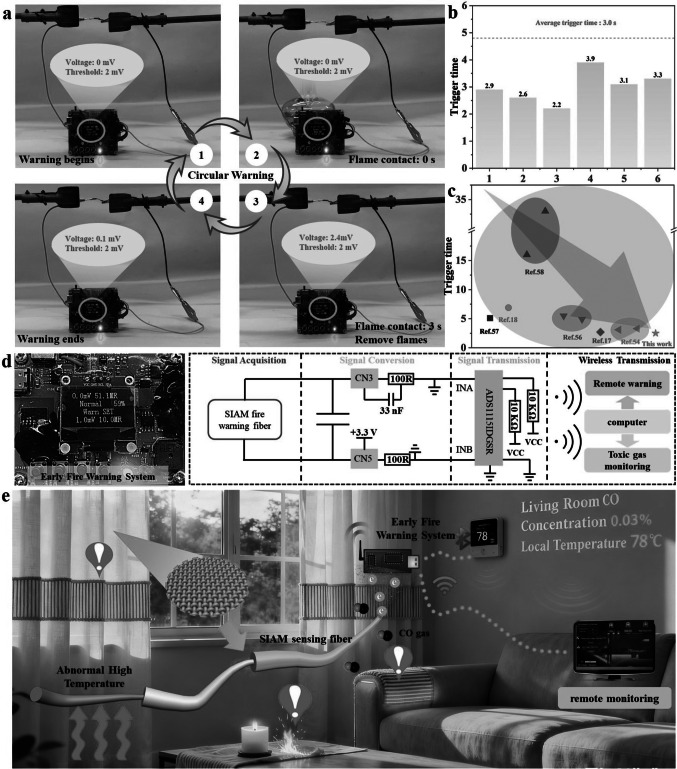
An early fire-warning system based on SIAM Sensing fiber. **a** Early fire-warning device based on SIAM sensing fiber. **b** SIAM sensors provide a warning time of 6 cycles. **c** Comparison of the warning time of SIAM sensing fibers with warning times in other literature. **d** Wireless fire alarm equipment and circuit diagram. **e** Application of SIAM sensing fibers in early fire warning

The aforementioned experiments have confirmed that the SIAM sensing fiber exhibits high sensitivity for both temperature sensing and CO gas sensing. Therefore, the SIAM sensing fiber can be utilized as an accurate and reliable sensor for temperature and CO gas, facilitating early fire warnings in residential settings. Based on a dual-channel (voltage and resistance) alarm device developed by our research team (Figs. S16 and S17), a wireless early fire-warning system was constructed by integrating the SIAM sensing fiber. This system can trigger an alarm without an external power source. As illustrated in Fig. 7a, it consists of an alarm device integrated with the SIAM sensing fiber. When the fiber is exposed to an alcohol lamp flame, a thermoelectric voltage is generated which is subsequently detected by the alarm device as an electrical signal. Once the thermoelectric voltage exceeds the device’s preset threshold of 2 mV, the alarm is activated, accompanied by a red warning light and a buzzer. Based on the calculations, this voltage corresponds to a temperature of approximately 150 °C, which is substantially higher than typical ambient temperatures and is therefore indicative of an abnormal temperature rise associated with potential fire hazards. As shown in Fig. 7b, this wireless early fire-warning system demonstrates sensitive alert functionality, triggering an alarm within approximately 3 s after exposure to the alcohol lamp flame (As shown in Movie S2). Moreover, the system supports repeated warning operations, highlighting its excellent stability. A comparison of the warning time between the wireless early fire-warning system developed in this study and those reported in previous works is presented in Fig. 7c. The warning response time of the system is significantly shorter, indicating its high sensitivity. The alarm device used in this study is depicted in Fig. 7d, which primarily includes modules for receiving and processing temperature-sensitive voltage signals and gas-sensitive resistance signals, along with an alarm unit. In the CO gas alarm experiment (Fig. S18), exposure of the SIAM sensing fiber to a specific concentration of CO gas leads to a rapid decrease in its electrical resistance. Once the resistance drop surpasses the set threshold, the warning light and buzzer in the alarm unit are activated. As illustrated in Fig. 7e, the SIAM sensing fiber can be attached or woven into curtains and other household furnishings. The wireless alarm system enables accurate detection of ambient temperature and CO gas concentration, providing timely alerts at the early stages of a fire. Furthermore, the system supports wireless transmission via Bluetooth or Wi-Fi to mobile devices, allowing remote monitoring of the indoor environment. Even in the absence of occupants, potential fire hazards can be promptly detected via mobile devices, thereby enabling comprehensive, timely early fire warning.

## Conclusion

In summary, we present a temperature-CO bimodal early fire-warning sensing fiber with gradient porous sheath structure through coaxial wet spinning combined with gradient-induced phase separation technology. The gradient pore structure of the fiber sheath achieves a transition from larger outer pores to gradually smaller inner pores. This architecture enables rapid diffusion of CO gas into the fiber interior and full combination with adsorption sites. By incorporating SnO_2_/In_2_O_3_ heterojunctions in the fiber sheath, the fiber demonstrates fast CO response (19.28 s) and high sensitivity (0.95%/ppm), representing a 15% sensitivity improvement compared to non-gradient porous fibers. The NH_4_^+^ crosslinked MXene conductive network in the fiber core layer enables precise temperature sensing within the 50–300 °C range, owing to its outstanding thermoelectric performance (Seebeck coefficient of 20.6 *μ*V K^−1^). Additionally, an ANFs isolation layer is incorporated between the fiber core and sheath layers, effectively preventing mutual interference between temperature sensing signals and gas-sensing signals, thereby successfully establishing dual temperature-gas detection channels within a single fiber. In subsequent tests, the fiber demonstrated rapid flame warning capabilities (3 s response time) and accurate CO gas monitoring performance. Furthermore, the fiber exhibits excellent flame retardancy (LOI = 41.5%), effectively ensuring its stability and repeatability during monitoring processes. Finally, the resultant SIAM sensing fiber was connected to a self-made wireless early fire-warning system, achieving a rapid response to fire in ~ 3 s and detecting 10 ppm of CO gas within 19 s. This work paves a promising pathway for developing highly sensitive and reliable dual-parameter early fire-warning sensors.

## Supplementary Information

Below is the link to the electronic supplementary material.

Supplementary file1 (MP4 8325 KB)

Supplementary file2 (MP4 5432 KB)

Supplementary file3 (DOCX 3296 KB)
