# Supplementary material for: Biomimetic Gradient Porous Core–Shell Fibers with Enhanced Gas Sensing for CO-Temperature Dual-Mode Early Fire Warning
Source: Nanomicro Lett. 2026 Aug 3;19:17. doi: 10.1007/s40820-026-02292-8 (PMC13429571; doi:10.1007/s40820-026-02292-8)
Supplement: Supplementary file 3 — Supplementary file3 (DOCX 3296 KB) [file 40820_2026_2292_MOESM3_ESM.docx]

Supporting Information for

**Biomimetic Gradient Porous Core–Shell Fibers with Enhanced Gas Sensing for CO-Temperature Dual-Mode Early Fire Warning**

Lele Huang ^1^, Xingyu He ^1^, Jianan Jiang ^1^, Xiaoqian Li ^1^, Mi Zhou ^1^, Md Hasib Mia ^2^, Min Hong ^3^, Hualing He ^1,^ **^*^**, Siqi Huo ^3^**^,^** **^*^**, Zhicai Yu ^1^**^,^** **^*^**

^1^ State Key Laboratory of New Textile Materials and Advanced Processing, Hubei Key Laboratory of Biomass Fibers and Eco-dyeing & Finishing, School of Textile Science and Engineering, Wuhan Textile University, Wuhan 430200, P. R. China

^2^ Key Laboratory of High Performance fibers & products, Ministry of Education, College of Materials Science and Engineering, Donghua University, Shanghai 201620, P. R. China

^3^ Centre for Future Materials, School of Science, Engineering and Digital Technologies, University of Southern Queensland, Springfield Central 4300, Australia

*****Corresponding authors**.** E-mail: [hehualinghe@126.com](mailto:hehualinghe@126.com) (Hualing He); [Siqi.Huo@unisq.edu.au](mailto:Siqi.Huo@unisq.edu.au) or [sqhuo@hotmail.com](mailto:sqhuo@hotmail.com) (Siqi Huo); [yuzhicaicai@163.com](mailto:yuzhicaicai@163.com) (Zhicai Yu)

**S1 Supplementary Text**

***S1.1 Preparation of Ti_3_C_2_T_X_ MXene Nanosheets***

Ti_3_C_2_T_x_ MXene nanosheets were synthesized by selectively etching the aluminum layer from Ti_3_AlC_2_ MAX phase powder using an in-situ generated LiF/HCl etchant. Specifically, LiF (1.6 g) was dissolved in 20 mL of concentrated HCl (12 mol/L) under magnetic stirring for 30 minutes. Subsequently, Ti3AlC2 powder (1 g) was gradually added to the etching solution in small portions. The reaction mixture was magnetically stirred at room temperature for 24-36 hours to ensure complete etching of the Al layer. The resulting MXene nanosheets were repeatedly washed via centrifugation with distilled water at 4000 rpm until the supernatant reached a neutral pH. The collected sediment was delaminated for 2 hours, followed by freeze-drying to obtain single-layer MXene nanosheets. A 50 mg/mL aqueous dispersion of the obtained MXene nanosheets was prepared and used as the core-layer spinning dope for the SIAM fibers.

***S1.2 Preparation of silver nanowires***

Ag nanowires (NWs) were synthesized via a conventional polyol process [S1]. In a typical procedure, 0.01 g of NaCl and 0.5 g of PVP were dissolved in 60 mL of ethylene glycol under magnetic stirring at 700 r/min for 15 min. Subsequently, 0.51 g of AgNO₃ was introduced into the mixture and further stirred for 5 min. The resulting solution was then transferred into a PTFE-lined stainless steel autoclave and heated in an oven at 180 °C for 8 h. After the reaction, the system was allowed to cool naturally to room temperature. Finally, the product was purified through repeated centrifugation with ethanol at 3000 r/min and dried at 60 °C, yielding the final Ag NWs.

***S1.3 SnO_2_/In_2_O_3_ composite***

A certain amount of SnO_2_ was dissolved in deionized water, followed by the addition of In_2_O_3_ into the above SnO_2_ dispersion. The mass ratios of In_2_O_3_ to SnO_2_ were set as 2:10, 4:10, 6:10, 8:10, and 10:10, respectively. The mixed solution of SnO_2_ and In_2_O_3_ was then ultrasonicated for 2 h to achieve uniform dispersion. Subsequently, the well-dispersed mixture was dried in an oven at 100 ℃. The resulting solid was collected and ground into a fine powder to obtain the SnO_2_/In_2_O_3_ composite material.

**S2 Supporting Figures**


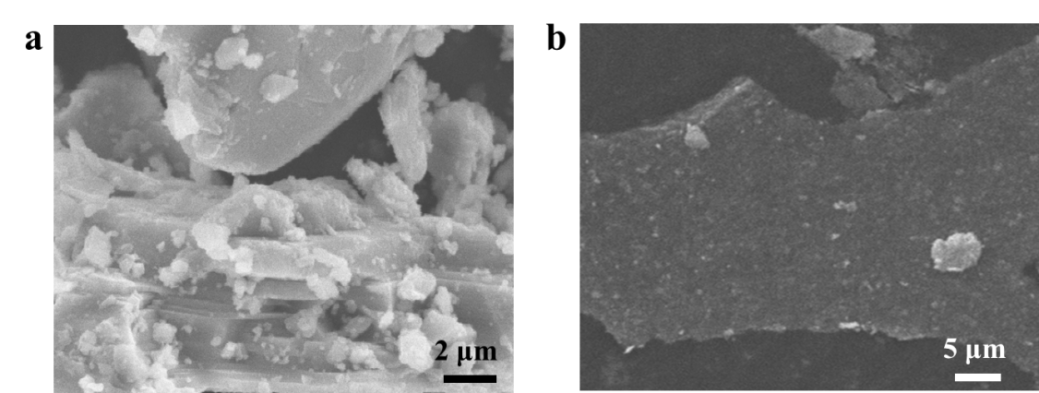


**Fig. S1 a** SEM of Ti_3_C_2_T_X_. **b** SEM of Mxene


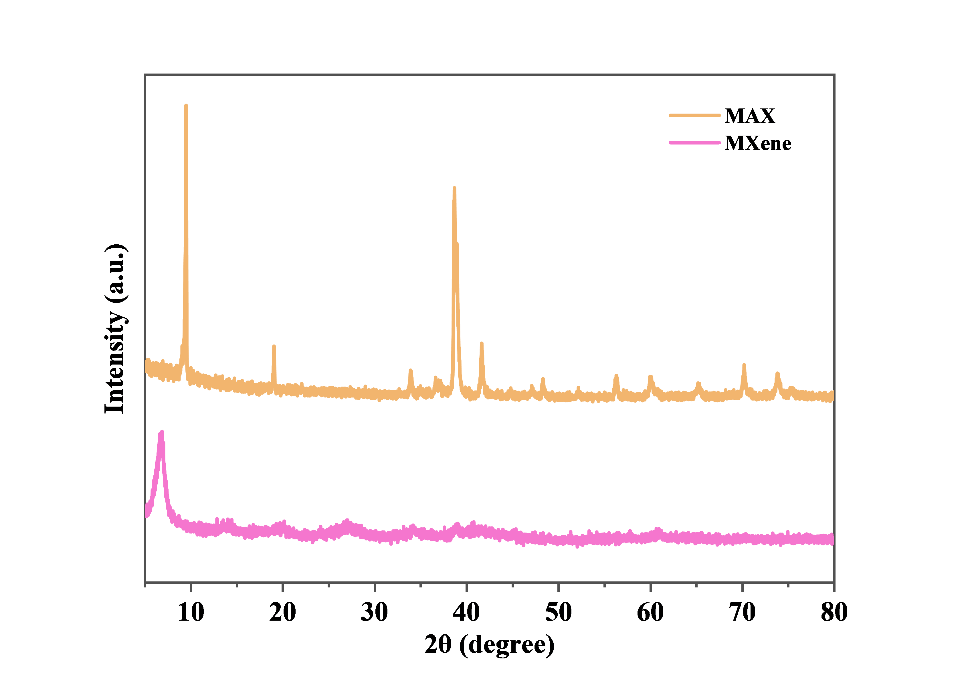


**Fig. S2** Ti_3_C_2_T_X_ and MXene XRD spectra


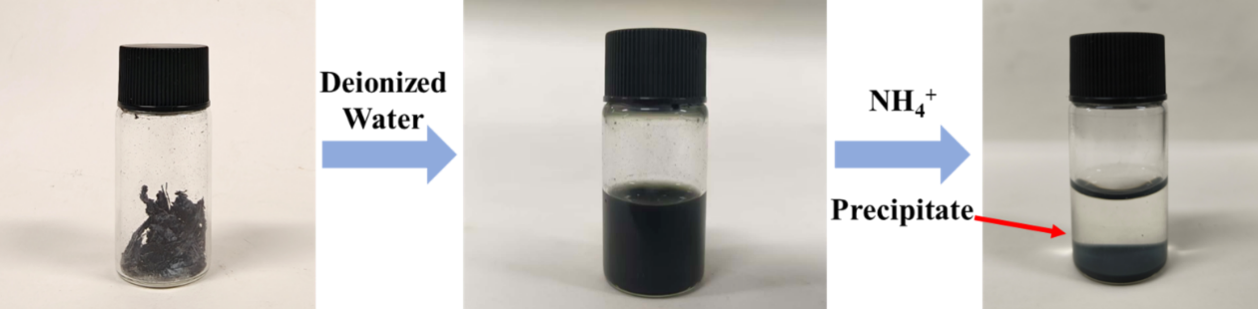


**Fig. S3** Cross-linking effect of NH_4_^+^ on MXene dispersion


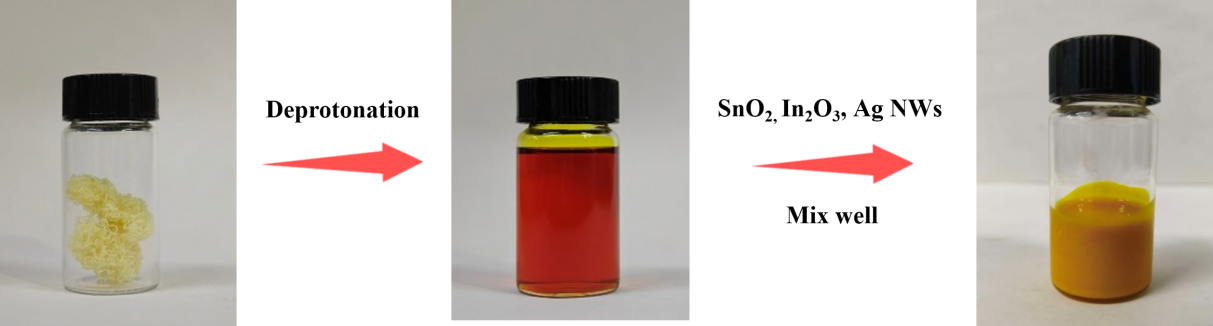


**Fig. S4** Preparation of spinning solution for the outermost layer of SIAM sensing fiber


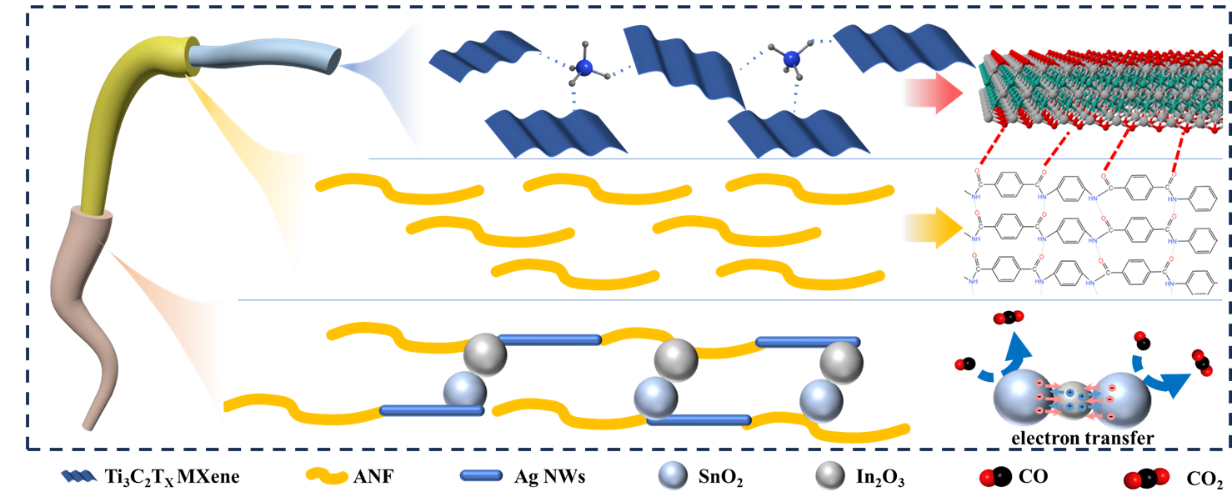


**Fig. S5** Schematic diagram of the three-layer structure of SIAM sensing fiber


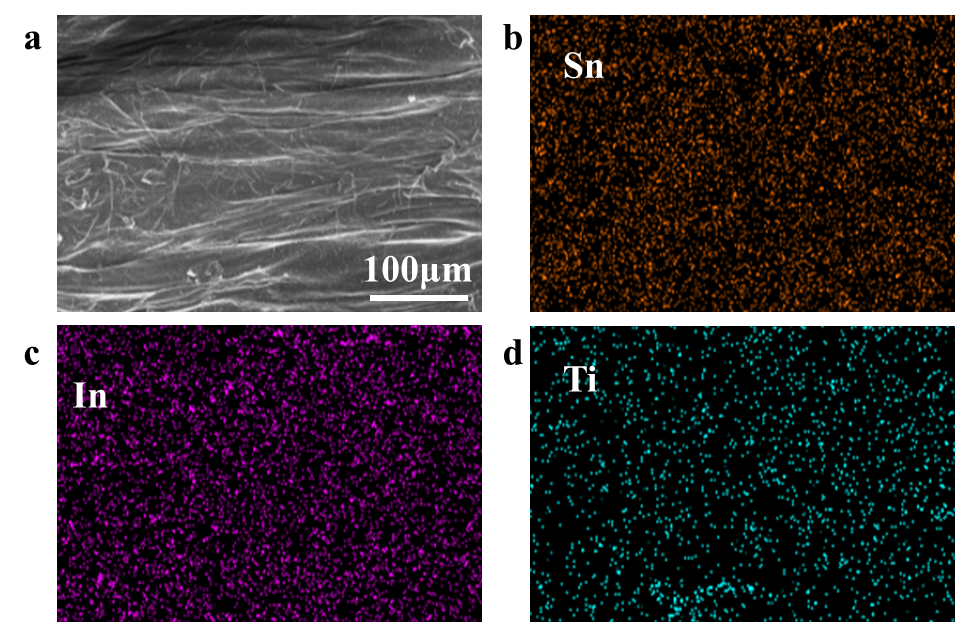


**Fig. S6 a** SEM images of SIAM sensing fibers. **b-d** The energy spectrum of the corresponding element


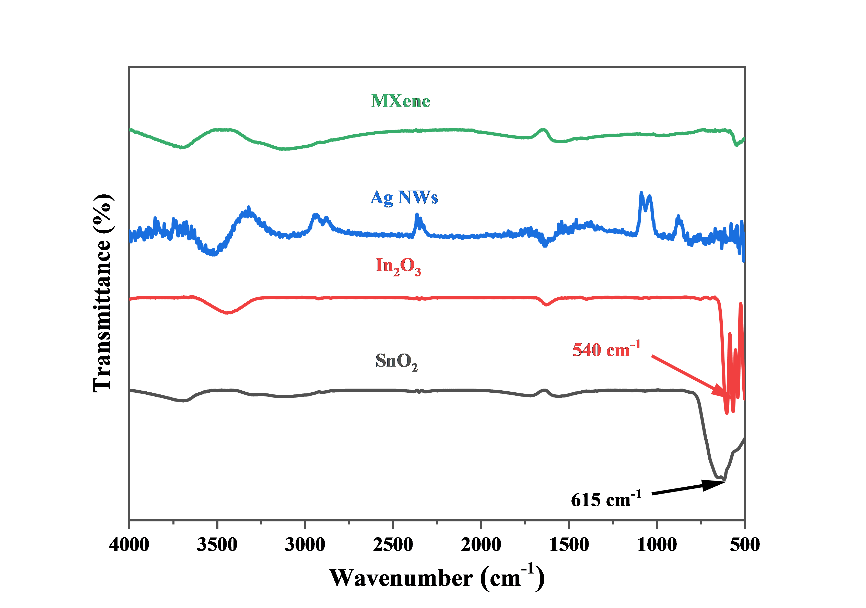


**Fig. S7** FTIR spectra of individual material components in SIAM sensing fibers


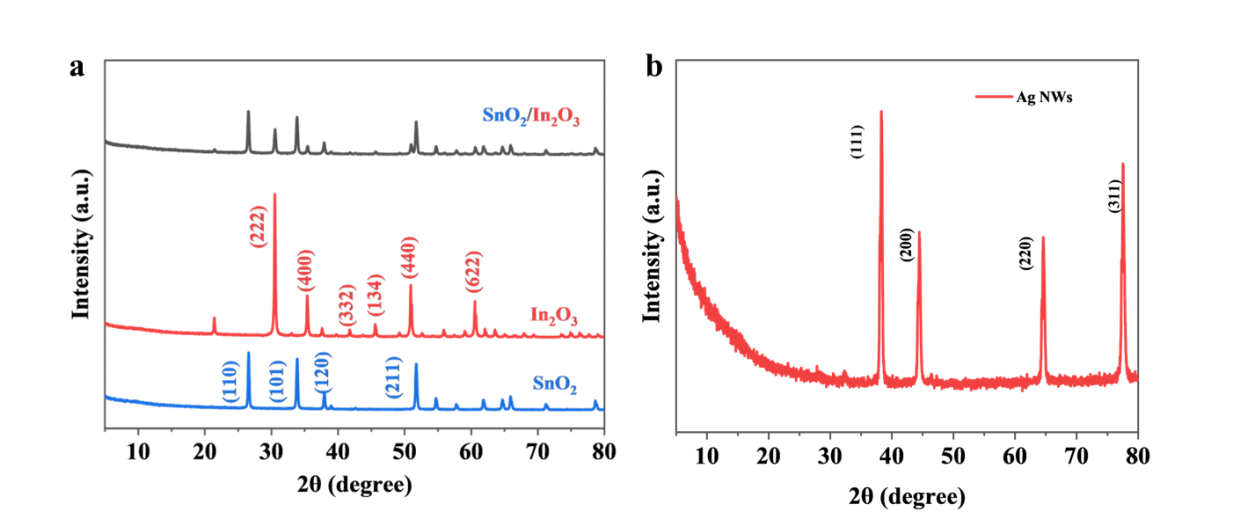


**Fig. S8** **a** XRD spectrum of SnO_2_, In_2_O_3_ and SnO_2_/In2O_3_ composites. **b** XRD spectra of Ag NWs


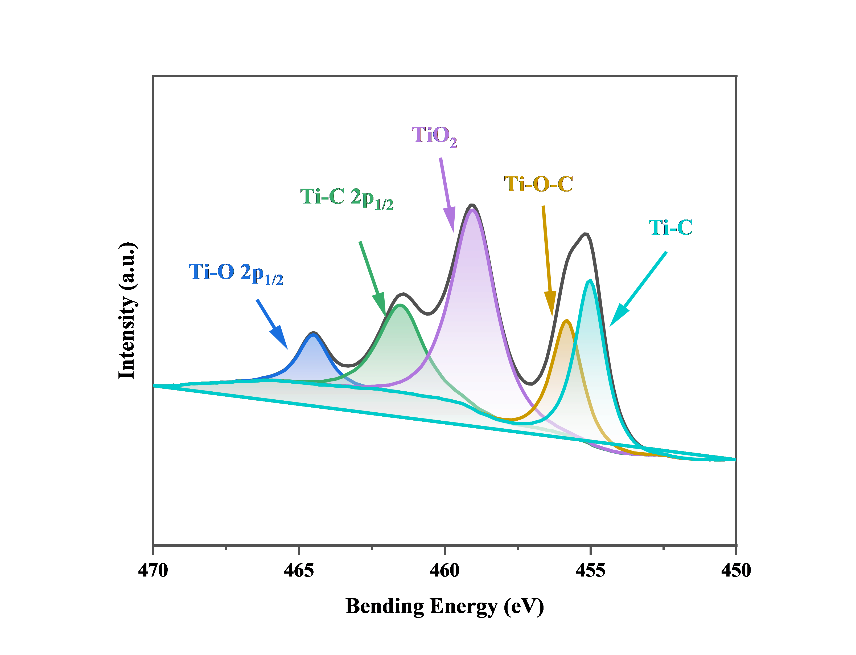


**Fig. S9** Fine spectral characterization of Ti element in XPS spectra of SIAM sensing fibers


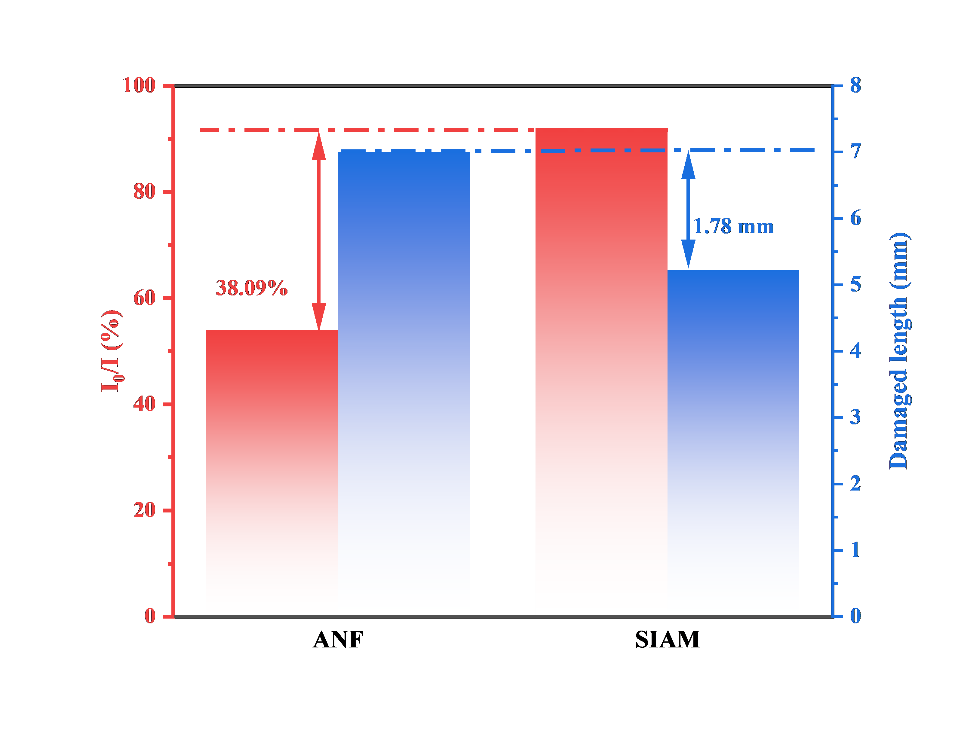


**Fig. S10** Flame Contact Test Results of SIAM Sensing Fiber


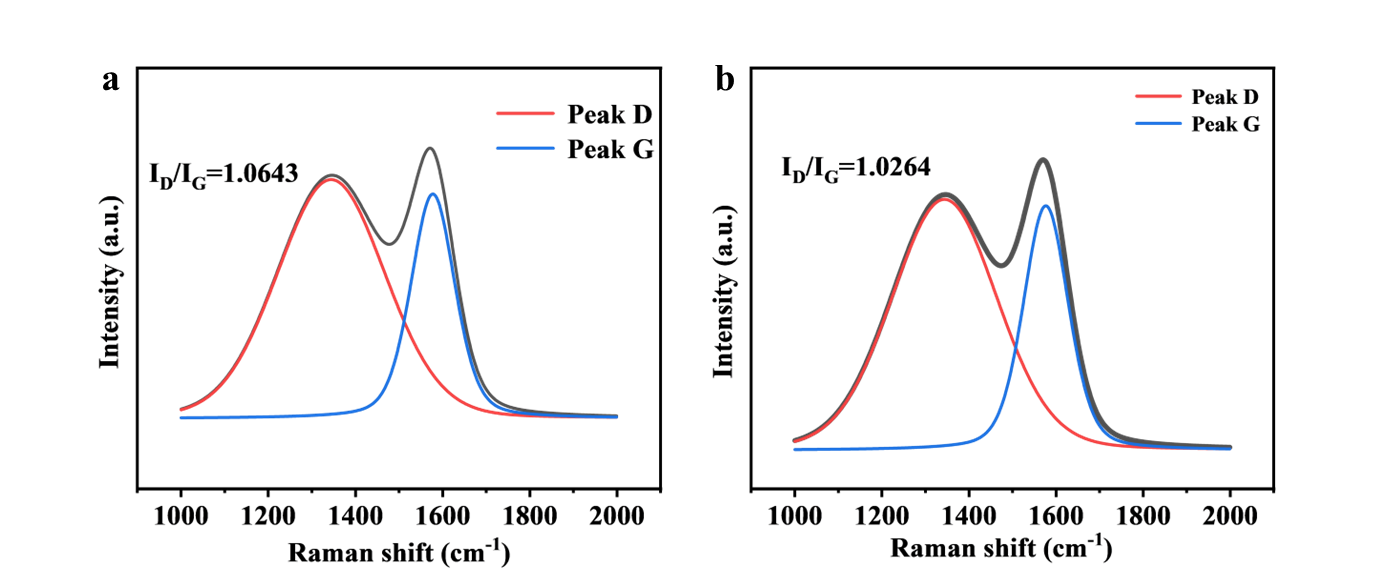


**Fig. S11 a** Raman spectra of ANF fibers after combustion. **b** Raman spectra of SIAM sensing fibers after combustion


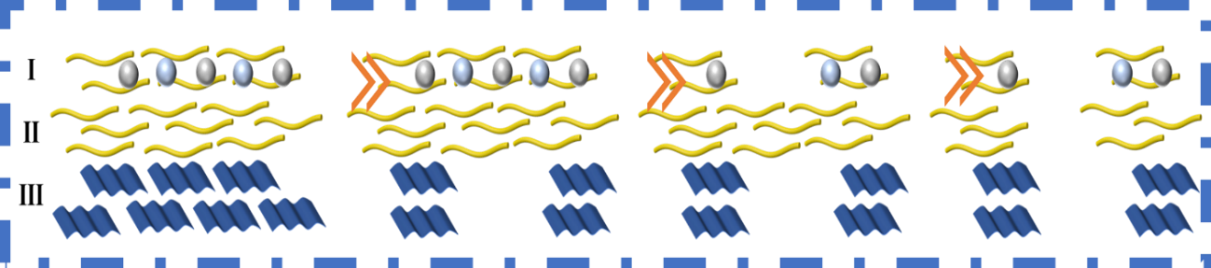


**Fig. S12** The fracture process of SIAM sensing fibers. I is the surface layer, II is the intermediate isolation layer, and III is the core layer


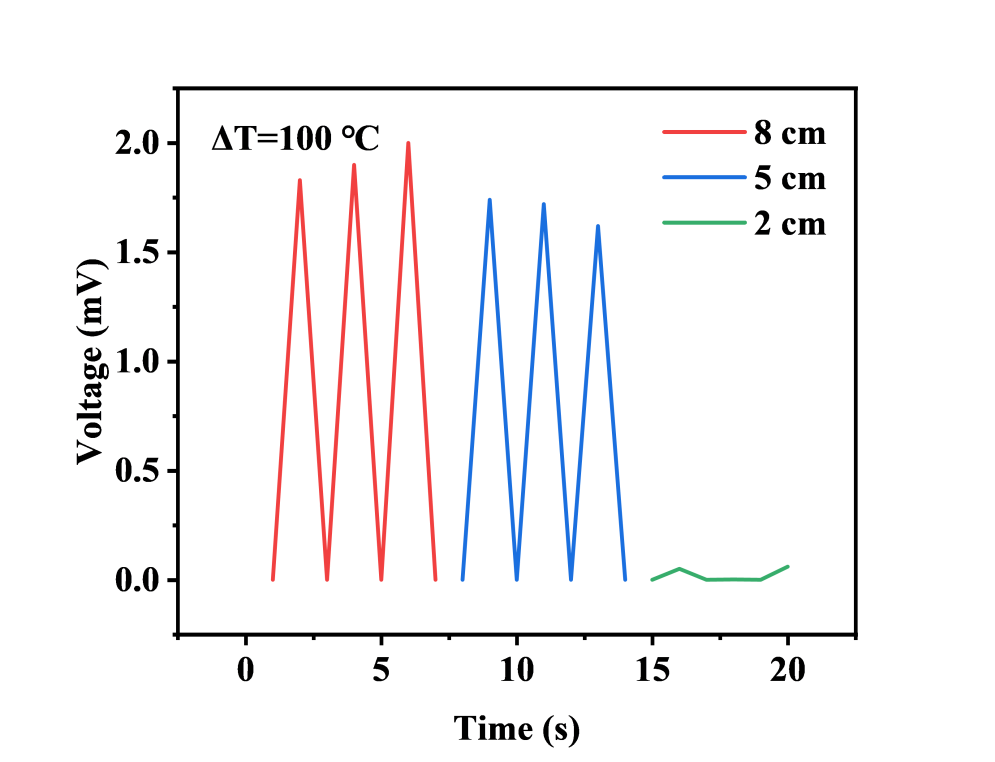


**Fig. S13** The relationship between the distance between the cold and hot ends of SIMA sensing fibers and the output voltage


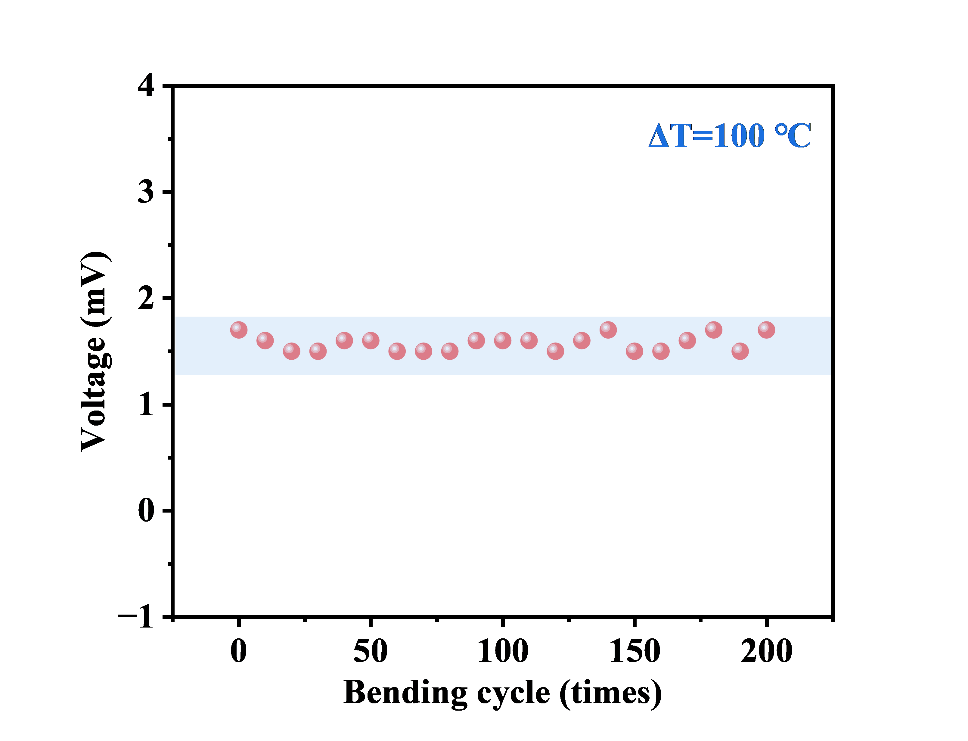


**Fig. S14** Output voltage of SIAM sensing fiber after multiple bending at a temperature difference of 100 ℃


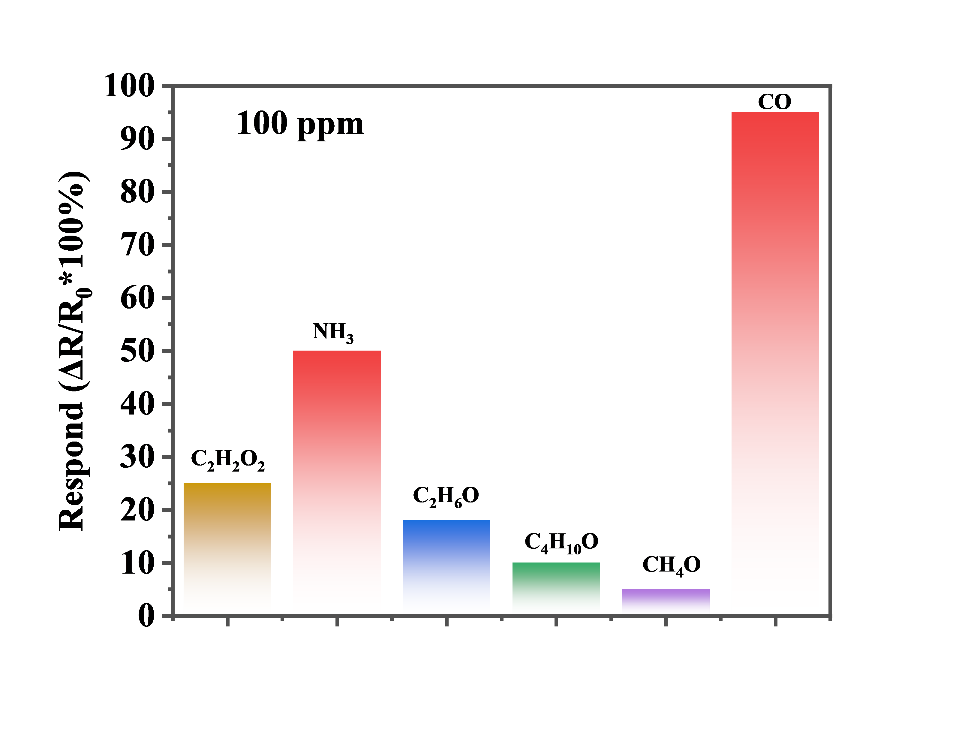


**Fig. S15** Comparison of Gas Selectivity of SIAM Sensing Fibers


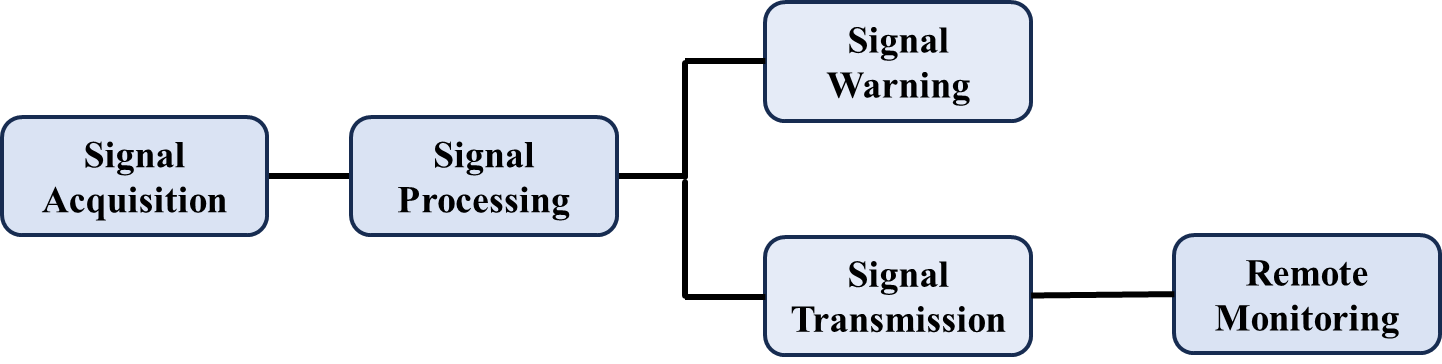


**Fig. S16** Dual-channel alarm device for voltage and resistance


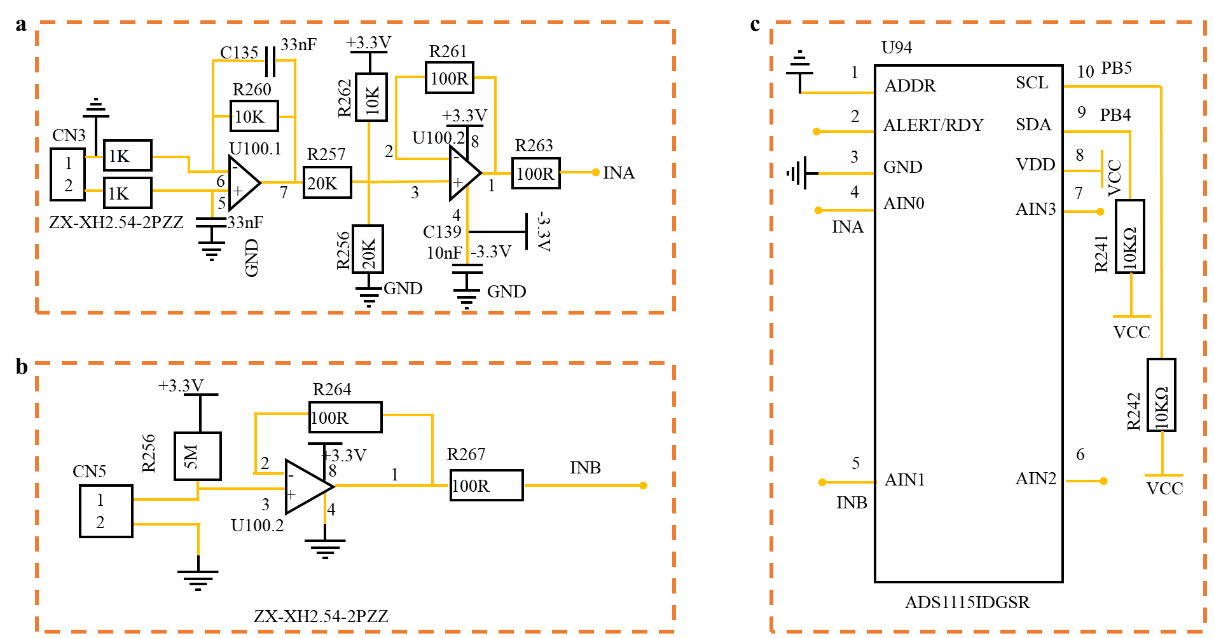


**Fig. S17** Internal wiring diagram of dual-channel alarm device for voltage and resistance. **a** Voltage signal processing, acquisition, and processing device. **b** Resistance signal processing, acquisition, and processing device. **c** Signal processing and output device


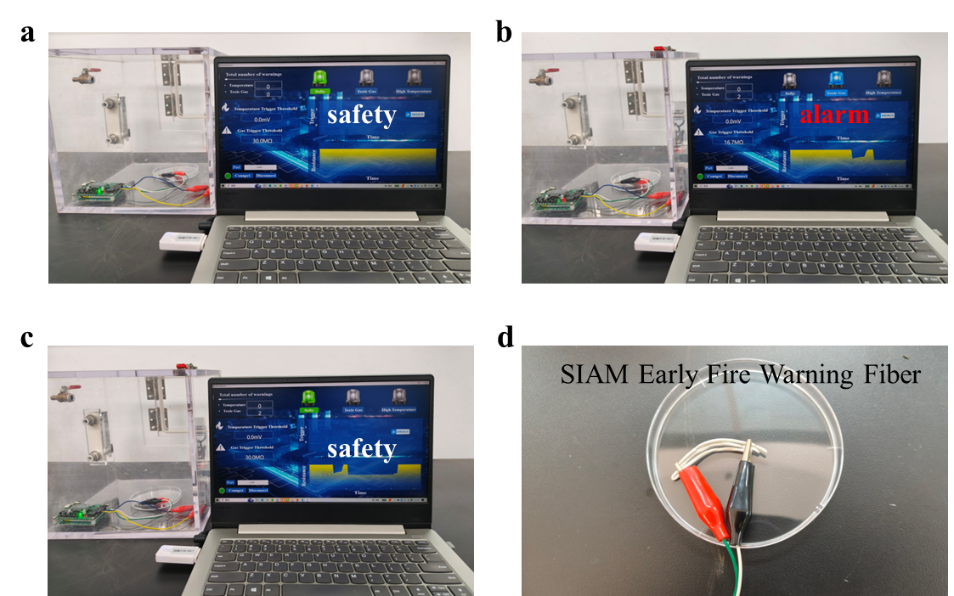


**Fig. S18** CO gas warning for wireless fire alarm system based on SIAM sensing fiber. **a** When CO is not introduced. **b** When CO is introduced. c. After excluding CO. d. SIAM sensing fiber

**S3 Supporting Tables**

**Table S1** Comparative Performance of CO Gas Sensing

| **Sample** | **Response time** | **Sensitivity** | **Temperature** |
| --- | --- | --- | --- |
| **Pt/SnO_2_ PNS [S2]** | 44 | 80 | 25 |
| **Zn/In_2_O_3_ [S3]** | 53 | 80 | 50 |
| **Pd/SnO_2_ [S4]** | 40 | 50 | 100 |
| **Pd-Al_2_O_3_/SnO_2_ [S5]** | 50 | 70 | 100 |
| **This work** | 19 | 95 | 25 |

**S4 Supporting Movies**

**S4.1 Movie S1** Vertical combustion test of SIAM sensing fiber.

**Description:** Use a fixture to clamp one end of the SIAM sensing fiber (approximately 10cm in length), with the other end of the fiber naturally vertically downward. Afterwards, ignite the alcohol lamp and place it under the fiber. Burn one end of the SIAM sensing fiber with the flame of the alcohol lamp. After being burned for a certain period of time, the fiber surface turned black and no melting or dripping occurred. This indicates that SMA fibers have good flame-retardant properties.

**S4.2 Movie S2** Fire warning testing of SIAM sensing fibers.

**Description:** Connect the SIAM sensing fiber core layer to the wireless fire alarm device through wires to form a wireless early fire alarm system. When the SIAM sensing fiber is exposed to the flame of the alcohol lamp, the alarm light quickly triggers within 3 seconds. Indicating that SIAM sensing fibers have sensitive fire alarm response performance.

**Supplementary References**

[S1] H. He, Y. Qin, J. Liu, Y. Wang, J. Wang et al., A wearable self-powered fire warning e-textile enabled by aramid nanofibers/MXene/silver nanowires aerogel fiber for fire protection used in firefighting clothing. Chem. Eng. J. **460**, 141661 (2023). <https://doi.org/10.1016/j.cej.2023.141661>

[S2] Y. Li, X. Song, L. Li, W. Wu, K. Tao et al., Low concentration CO gas sensor constructed from MoS_2_ nanosheets dispersed SnO_2_ nanoparticles at room temperature under UV light. Ceram. Int. **49**, 10249–10254 (2023). <https://doi.org/10.1016/j.ceramint.2022.11.204>

[S3] N. Singh, C. Yan, P. Lee. Room temperature CO gas sensing using Zn-doped In_2_O_3_ single nanowire field effect transistors. Sens. Actuators B **150**, 19–24 (2010). <https://doi.org/10.1016/j.snb.2010.07.051>

[S4] K. Wang, T. Zhao, G. Lian, Q. Yu, C. Luan et al., Room temperature CO sensor fabricated from Pt-loaded SnO_2_ porous nanosolid. Sens. Actuators B **184**, 33–39 (2013). <https://doi.org/10.1016/j.snb.2013.04.054>

[S5] H. Zhang, Y. Long, Z. Li, B. Sun. Fabrication of comb-like ZnO nanostructures for room-temperature CO gas sensing application. Vacuum **101**, 113–117 (2014). <https://doi.org/10.1016/j.vacuum.2013.07.046>
